# Molecular basis for RASSF10/NPM/RNF2 feedback cascade–mediated regulation of gastric cancer cell proliferation

**DOI:** 10.1016/j.jbc.2021.100935

**Published:** 2021-07-03

**Authors:** Naga Padma Lakshmi Ch, Ananthi Sivagnanam, Sebastian Raja, Sundarasamy Mahalingam

**Affiliations:** Laboratory of Molecular Cell Biology, National Cancer Tissue Biobank, Department of Biotechnology, Bhupat and Jyoti Mehta School of Biosciences, Indian Institute of Technology-Madras, Chennai, India

**Keywords:** RASSF10, cell proliferation, mitosis, proteomics, nucleophosmin, ubiquitination, RNF2, GADD45a, gastric cancer, MS, APC, anaphase-promoting complex, BrdU, bromodeoxyuridine, CHX, cycloheximide, GADD45a, growth arrest and DNA damage–inducible alpha, MTT, 3-(4,5-dimethylthiazol-2-yl)-2,5-diphenyltetrazolium bromide, NPM, nucleophosmin, RASSF, Ras-association domain family, RNF2, ring finger protein 2, TCGA, the cancer genome atlas

## Abstract

Ras-association domain family (RASSF) proteins are encoded by numerous tumor suppressor genes that frequently become silenced in human cancers. RASSF10 is downregulated by promoter hypermethylation in cancers and has been shown to inhibit cell proliferation; however, the molecular mechanism(s) remains poorly understood. Here, we demonstrate for the first time that RASSF10 inhibits Cdk1/cyclin-B kinase complex formation to maintain stable levels of cyclin-B for inducing mitotic arrest during cell cycle. Using LC-MS/MS, live cell imaging, and biochemical approaches, we identify Nucleophosmin (NPM) as a novel functional target of RASSF10 and revealed that RASSF10 expression promoted the nuclear accumulation of GADD45a and knockdown of either NPM or GADD45a, resulting in impairment of RASSF10-mediated G2/M phase arrest. Furthermore, we demonstrate that RASSF10 is a substrate for the E3 ligase ring finger protein 2 (RNF2) and show that an NPM-dependent downregulation of RNF2 expression is critical to maintain stable RASSF10 levels in cells for efficient mitotic arrest. Interestingly, the Kaplan–Meier plot analysis shows a positive correlation of RASSF10 and NPM expression with greater gastric cancer patient survival and the reverse with expression of RNF2, suggesting that they may have a role in cancer progression. Finally, our findings provide insights into the mode of action of the RASSF10/NPM/RNF2 signaling cascade on controlling cell proliferation and may represent a novel therapeutic avenue for the prevention of gastric cancer metastasis.

*RAS* oncogenes are central players in many human cancers. Ras regulates various physiological functions through downstream molecules known as Ras effectors (1). In the past decade, a distinct class of nonenzymatic Ras effectors known as Ras-association domain family (RASSF) of proteins that are characterized by the presence of Ras-association domain (Ral guanine nucleotide dissociation stimulator and ALL-1 fusion partner from chromosome 6) has been identified (2). The RASSF consists of ten members, and based on the location of the Ras-association domain, they are subdivided into two groups namely classical RASSFs, also known as C-terminal RASSFs (RASSF1-6) and N-terminal RASSFs (RASSF7-10) (3). Most of the RASSF members are known to be downregulated in various human cancers by epigenetic modifications (3). RASSF10 is a member of the N-terminal RASSFs. *RASSF10* gene is located on chromosome 11p15.2 and has a CpG island of >2 Kb in its promoter region and encodes a protein of 507 amino acids. RASSF10 is normally expressed in a wide variety of tissues including the brain, thyroid, pancreas, placenta, heart, lung, and kidney (4). Expression of RASSF10 was known to be downregulated by promoter hypermethylation across several cancers (4). Cellular distribution of RASSF10 appears to be cell cycle dependent (5). Association of RASSF10 with centrosomes/microtubules during mitosis is critical to regulate cell viability, cell proliferation, migration and to increase the efficiency of microtubule inhibitor drugs (5, 6). These data suggest that RASSF10 might play an important role in mitotic phase regulation during cell cycle. The tumor suppressor role of RASSF10 has been described in several types of cancers (5, 7, 8). However, the molecular mechanism(s) by which RASSF10 executes its function during cell proliferation and survival is poorly understood.

Downregulation of RASSF10 expression has been associated with poor survival of patients with gastric cancer (7). RASSF10 has been reported to modulate Wnt/β-catenin signaling and Jun N-terminal kinase /c-Jun/AP-1 pathway to regulate gastric cancer progression (9). Consistently, a recent report suggests that the status of RASSF10 promoter methylation may serve as a valuable indicator for the diagnosis and prognosis of gastric cancer (10). Together, these reports suggest that RASSF10 might be regulating a delicate network of pathway(s) to control cell proliferation and survival during cancer progression. We therefore attempted to explore the mechanism by which RASSF10 regulates cell proliferation and survival using gastric cancer as a model system. In the present investigation, using proteomics, nucleophosmin (NPM) was identified as a novel functional target of RASSF10. Furthermore, RASSF10 promotes G2/M phase arrest during cell division cycle by inhibiting the complex formation between cyclin-B and CDK1 in NPM-dependent manner. In addition, NPM promotes RASSF10 stabilization by altering the expression of E3 ligase RING2 (RNF2), which is critical for controlling cell proliferation during tumorigenesis.

## Results

### RASSF10 induces G2/M phase arrest of the cell division cycle

**Figure 1:**
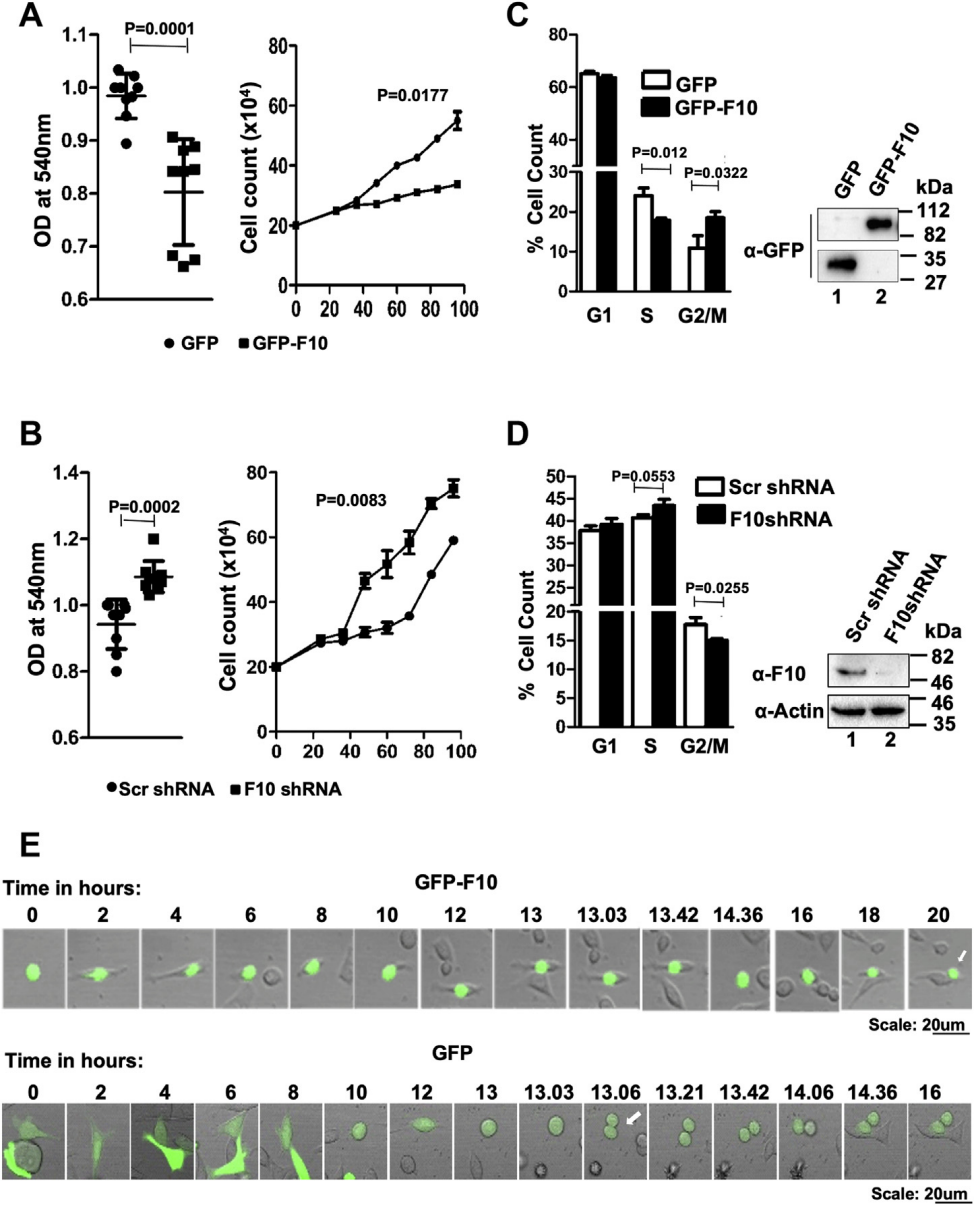
**RASSF10 induces G2/M phase arrest of the cell division cycle.** RASSF10 was ectopically expressed (*A*) or depleted (*B*) by specific shRNA in AGS cells; MTT assay was performed for cell viability, and cell numbers at different time intervals were counted for cell proliferation. Results suggest that RASSF10 expression significantly reduced cell viability as well as proliferation, and the reverse was observed under knockdown conditions. *C*, ectopic expression of RASSF10 arrest cells at the G2/M phase of the cell cycle. *D*, results from the cell cycle analysis suggest that RASSF10 knockdown promoted faster mitotic exit. *E*, ectopic expression of RASSF10 inhibits cell division. AGS cells were synchronized at the G1 stage by double thymidine block, and the cell division cycle was analyzed using live cell imaging as described in Experimental procedures. F10, RASSF10; MTT, 3-(4,5-dimethylthiazol-2-yl)-2,5-diphenyltetrazolium bromide.

RASSF10 expression was downregulated in a wide range of cancers because of promoter hypermethylation (5, 7, 8). A recent report suggests that RASSF10 induces apoptosis and reduces cell proliferation by modulating the cell cycle (11), but the underlying mechanism(s) remains elusive. To this end, combination of 3-(4,5-dimethylthiazol-2-yl)-2,5-diphenyltetrazolium bromide (MTT), measurement of cell numbers, bromodeoxyuridine (BrdU) incorporation assays, and flow cytometric analysis were performed with perturbations of RASSF10 expression levels in AGS cells. Results from MTT, cell number measurement at different time points (Fig. 1*A*), and BrdU (Fig. S1*A*) incorporation assays indicated that RASSF10 reduces cell proliferation. In contrast, increased cell proliferation was observed when RASSF10 was depleted with specific shRNA (Fig. 1*B* and Fig. S1*B*). Consistently, results from flow cytometry analysis suggest that ectopic expression of RASSF10 resulted in accumulation of more cells at the G2/M phase of the cell division cycle (Fig. 1*C*) and was reversed under RASSF10 knockdown condition (Fig. 1*D*). RASSF10 protein levels were determined using anti-GFP (Fig. S1, *A* and *C* and Fig. 1*C*) or anti-RASSF10 (Fig. S1, *B* and *D* and Fig. 1*D*) antibodies. To confirm the above observation, live cell imaging analysis was performed with AGS cells expressing RASSF10 that were synchronized at the G1/S phase with double thymidine block (Fig. S1, *E* and *F*), as described in Experimental procedures. Interestingly, RASSF10-expressing cells were undivided even 20 h after release from double thymidine block (Fig. 1*E*; upper panel and Video S1), which suggests a mitotic arrest. In contrast, GFP-transfected cells divided 13 h after thymidine block release (Fig. 1*E*; lower panel and Video S2). Together, these results suggest that RASSF10 suppresses cell proliferation by regulating the G2/M phase of the cell division cycle.

### RASSF10 impedes mitotic exit by inhibiting Cdk1–cyclin-B kinase complex formation

**Figure 2:**
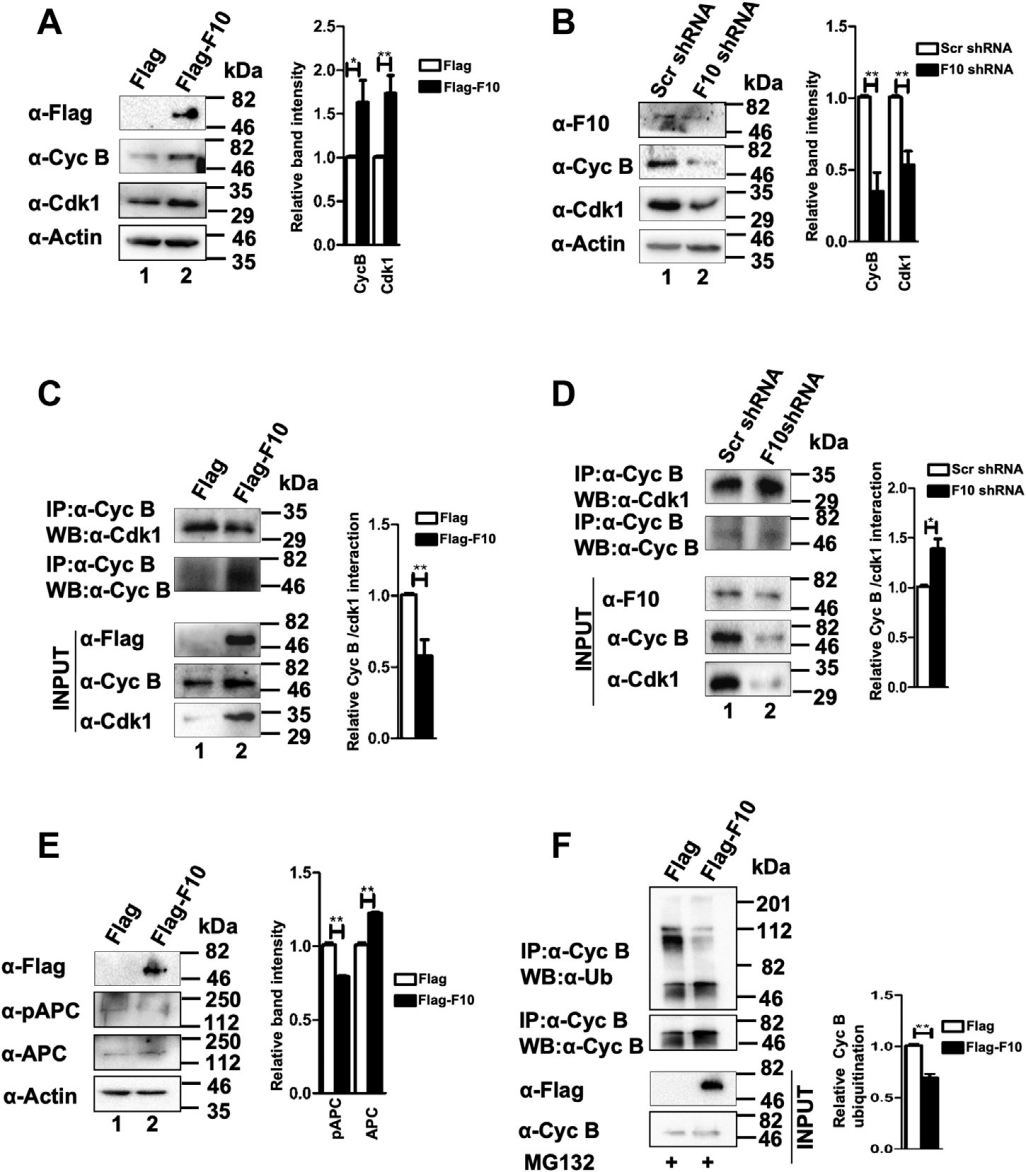
**RASSF10 impedes mitotic exit by inhibiting cyclin-B–Cdk1 kinase complex formation.***A*, RASSF10 upregulates the expression of cyclin-B and Cdk1 in AGS cells. *B*, knockdown of RASSF10 resulted in downregulation of cyclin-B and Cdk1 expression. *C*, ectopic expression of RASSF10 resulted in reduced cyclin-B–Cdk1 complex formation. *D*, RASSF10 depletion promoted cyclin-B–Cdk1 complex formation. *E*, RASSF10 abrogates APC1 phosphorylation despite increased expression of APC1 in AGS cells. *F*, ectopic expression of RASSF10 resulted in reduced ubiquitination of cyclin-B. The pulldown efficiency of cyclin-B was determined by stripping followed by probing the top panel using anti-cyclin-B antibody. The densitometry analysis of Western blots was carried out by normalizing the expression level of endogenous proteins to β-actin, as the loading control (n = 3, and data are expressed as the mean ± SD). APC1, anaphase-promoting complex 1; Cyc B, cyclin-B; F10, RASSF10; Ub, ubiquitin. (∗p<0.05; ∗∗p<0.01).

Activities of cyclins and cyclin-dependent kinases are critical to regulate cell cycle progression (12). To understand the mechanism of RASSF10-mediated G2/M phase arrest, we first measured the levels of cyclin-B, a mitotic cyclin, and its partner Cdk1. Interestingly, ectopic expression of RASSF10 resulted in upregulation of cyclin-B and Cdk1 protein levels (Fig. 2*A*); in contrast, knockdown of endogenous RASSF10 by shRNA significantly reduced the levels of cyclin-B and Cdk1 in AGS cells (Fig. 2*B*). It is well documented that the activity of the Cdk1–cyclin-B kinase complex is critical for mitotic progression (13). Together, these results lead to the hypothesis that RASSF10 may alter G2/M phase progression by modulating Cdk1–cyclin-B complex formation. To this end, the status of cyclin-B and Cdk1 complex formation was checked under ectopic expression and knockdown conditions of RASSF10. Results in Figure 2*C* reveal that ectopic expression of RASSF10 significantly reduced the levels of the Cdk1–cyclin-B complex (upper panel, lane 2) and the reverse was observed under RASSF10 knockdown conditions (Fig. 2*D*; upper panel, lane 2). These results were further confirmed with reverse coimmunoprecipitation using indicated antibodies in AGS cells (Fig. S2*A*). It is known that Cdk1–cyclin-B kinase phosphorylates anaphase-promoting complex (APC) at S^355^ and the activated APC ubiquitinates cyclin-B, which is critical for the initiation of mitotic exit (14, 15). Furthermore, we tested the status of APC phosphorylation and ubiquitination status of cyclin-B in presence of RASSF10. Interestingly, RASSF10 reduced APC phosphorylation despite increased expression of APC that was noticed in AGS cells (Fig. 2*E*; lane 2). Consistently, RASSF10 reduced the cyclin-B polyubiquitination (Fig. 2*F*) and the reverse was observed under RASSF10 knockdown conditions (Fig. S2*B*). Together, these results lead to the hypothesis that RASSF10 may interact with cyclin-B or Cdk1 to block the complex formation between cyclin-B and Cdk1. Surprisingly, the results from the coimmunoprecipitation experiment suggest that RASSF10 interacts with neither cyclin-B (Fig. S2*C*; lane 2) nor Cdk1 (Fig. S2*D*; lane 2). Results from the reverse coimmunoprecipitation experiment also confirmed the same (Fig. S2*E*). Collectively, these results suggest that RASSF10 inhibits APC activation by altering the complex formation between Cdk1 and cyclin-B to regulate mitotic exit.

### NPM is a novel functional target of RASSF10

**Figure 3:**
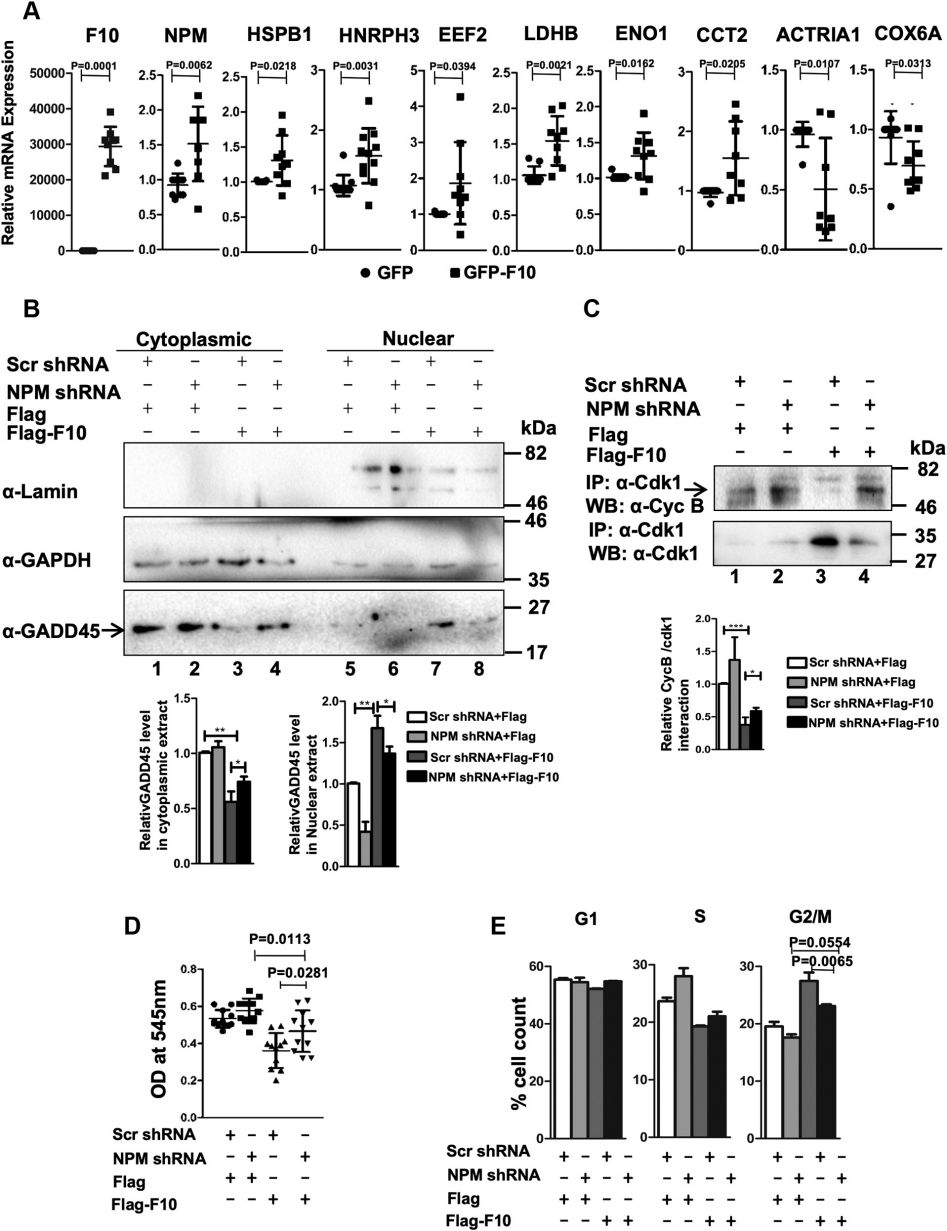
**NPM was identified as a novel functional target of RASSF10.***A*, RT-qPCR analysis suggests that expression of NPM, HSPB1, HNRPH3, EEF2, LDHB, ENO1, CCT2, ACTRIA1, and COX6A were deregulated by RASSF10. *B*, results from the subcellular fractionation followed by Western blot analysis suggest that RASSF10 promotes nuclear accumulation of GADD45a in NPM-dependent manner. *C*, results from the coimmunoprecipitation experiment suggest that NPM plays a critical role in RASSF10-mediated inhibition of cyclin-B–Cdk1 kinase complex formation. MTT and flow cytometry analyses suggest that RASSF10 failed to inhibit cell proliferation (*D*) and mitotic arrest (*E*) under NPM-knockdown condition. The densitometry analysis of Western blots was carried out by normalizing the expression level of endogenous proteins to the amount of protein used for pulldown, as the loading control (n = 3, and data are expressed as the mean ± SD). Cyc B, cyclin-B; GADD45a, growth arrest and DNA damage–inducible alpha; F10, RASSF10; MTT, 3-(4,5-dimethylthiazol-2-yl)-2,5-diphenyltetrazolium bromide; NPM, nucleophosmin. (∗*p* < 0.05; ∗∗*p* < 0.01; ∗∗∗*p* < 0.001).

We next performed an unbiased high-throughput two-dimensional electrophoresis/nLC-MS/MS to identify factor(s) that are critical for RASSF10 to modulate the Cdk1–cyclin-B kinase complex formation. Results from the MS analysis indicated that more than 45 proteins were significantly deregulated upon RASSF10 expression (Table S1). Interestingly, Protein ANalysis THrough Evolutionary Relationships (PANTHER) (16) indicated that 16 (53.3%) proteins were involved in the regulation of cell cycle and apoptosis, 9 (30%) in metabolic processes, 10 (33.4%) in biogenesis and biological regulation, cellular component organization, and biogenesis, 8 (26.6%) in response to stimulus and localization, and 3 (3.3%) in developmental, multicellular organismal, and signaling processes (Fig. S3*A*). To further confirm their expression status of target genes in presence of RASSF10, we have selected top 37 differentially regulated proteins based on protein identification score and percentage peptide coverage values. Results from the RT-qPCR analysis suggest that mRNA of *NPM*, *HSPB1*, *HNRNPH3*, *EEF2*, *LDHB*, *ENO1*, *CCT2*, *ACTRIA1*, and *COX6A* genes were significantly altered by RASSF10 (Table S2 and Fig. 3*A*). The transcript levels of NPM were consistently upregulated by RASSF10 with a more significant protein identification score and peptide coverage values from MS analysis (Table S1). Existing reports suggest that NPM-dependent nuclear translocation of growth arrest and DNA damage–inducible alpha (GADD45a) is critical to alter the complex formation between cyclin-B and Cdk1 (17, 18). Together, our results prompted us to hypothesize that the observed mitotic arrest might be due to RASSF10-induced NPM-dependent nuclear translocation of GADD45a followed by the alteration of Cdk1–cyclin-B kinase complex formation (Fig. S3*B*). To this end, we first checked the mRNA and protein levels of NPM under RASSF10 expression as well as knockdown conditions. Results from RT-qPCR and Western blot analyses indicated that both mRNA (Fig. S3*C*) and protein (Fig. S3*D*) levels of NPM were significantly upregulated by RASSF10 and the reverse was observed under knockdown condition (Fig. S3, *E* and *F*). The expression level of RASSF10 was determined by anti-GFP or anti-RASSF10 antibodies (Fig. S3, *D* and *F*). In addition, RASSF10-induced upregulation of the NPM protein level was further confirmed by two-dimensional electrophoresis followed by Western blot using the anti-NPM antibody (Fig. S4*A*), and the expression of RASSF10 was confirmed through Western blot analysis with the anti-GFP antibody (Fig. S4*B*). It is well documented that the function of NPM was dependent on its cellular localization (19); therefore, we next checked whether RASSF10 alters the NPM localization pattern in AGS cells. Results from immunofluorescence experiment indicated that nuclear localization of NPM was not altered by RASSF10 and interestingly both are localized in the nuclear compartment (Fig. S4*C*). Surprisingly, results from the coimmunoprecipitation analysis in AGS cells indicated that there is no interaction between RASSF10 and NPM, although both are localized to the nuclear compartment (Fig. S4*D*). These results were further confirmed with reverse coimmunoprecipitation (Fig. S4*E*). Collectively, these data provided convincing evidence that RASSF10 upregulates NPM expression by a novel mechanism.

To understand whether the RASSF10-mediated upregulation of NPM is responsible for the observed mitotic arrest, we immediately tested the subcellular distribution of GADD45a in presence of RASSF10 under NPM-depleted conditions. Results in Figure 3*B* indicate that RASSF10 promotes the nuclear accumulation of GADD45a protein (lower panel, lane 7). Interestingly, increased cytoplasmic retention of GADD45a was observed under the NPM knockdown condition with or without RASSF10 expression (Fig. 3*B*; lower panel, lanes 2 and 4). This was further confirmed by subcellular localization analysis using immunofluorescence assay (Fig. S5*A*). These data suggest that RASSF10 failed to promote nuclear accumulation of GADD45a in the absence of NPM although RASSF10 induces the expression of GADD45a (Fig. S5*B*; lanes 3 and 4). Surprisingly, we observed a reduction in RASSF10 protein levels under NPM knockdown conditions (Fig. S5*B*; upper panel, lane 4), and this experiment was repeated multiple times and observed similar results. Collectively, our results suggest that RASSF10-mediated nuclear retention of GADD45a is NPM dependent and further provided evidence that NPM is the potential functional target of RASSF10.

### NPM is critical for RASSF10-mediated mitotic arrest during cell division cycle

RASSF10 is known to stabilize p53 and modulates p53 signaling pathways during cell growth control (20). Interestingly, GADD45a is known to be highly expressed under stress conditions in p53-dependent and p53-independent manner (21). Together, these lead to the hypothesis that whether the RASSF10-mediated upregulation of GADD45a is p53 dependent? To confirm this, AGS cells were transfected with RASSF10 expression plasmids alone or in combination with shRNA specific to p53. Results from RT-qPCR analysis indicate that GADD45a mRNA levels were significantly upregulated by RASSF10 (Fig. S6*A*). Interestingly, Western blot analysis showed that RASSF10-mediated upregulation of GADD45a was abrogated under p53 knockdown conditions (Fig. S6*B*; lanes 3 and 4), which suggests that RASSF10 upregulates GADD45a expression in p53-dependent manner. To further understand whether p53 play any role in RASSF10-mediated nuclear translocation of GADD45a, we performed nuclear cytoplasmic fractionation followed by Western blot analysis in p53-deficient colon cancer cell line HCT116^*p53−/−*^ with RASSF10 expression under NPM knockdown conditions. Results in Fig. S6*C* clearly indicate the increased nuclear accumulation of GADD45a with RASSF10 expression (bottom panel; lane 7) as compared with the control (bottom panel; lane 5) although the absence of p53 and the reverse were observed in NPM-depleted cells (Fig. S6*C*; lane 8). Expression of RASSF10 and NPM was determined using anti-Flag and anti-NPM antibodies (Fig. S6*D*). Together, these data suggest that RASSF10-mediated GADD45a nuclear accumulation is NPM dependent but its expression is p53 dependent.

Recent report suggests that GADD45a interacts with Cdk1 and inhibits Cdk1–cyclin-B complex formation (18). To confirm whether RASSF10-induced NPM plays a role in altering Cdk1–cyclin-B complex formation to promote mitotic arrest, RASSF10 was expressed under NPM-knockdown conditions in AGS cells and the status of Cdk1–cyclin-B complex was checked using coimmunoprecipitation followed by Western blot analysis. Results in Figure 3*C* reveal that cyclin-B failed to form the complex with Cdk1 in cell lysates containing RASSF10 (upper panel; lane 3) despite increased expression of cyclin-B and Cdk1 (Fig. S7*A*; lane 3). In contrast, formation of the Cdk1–cyclin-B complex was noticed in cells with RASSF10 expression under NPM-knockdown conditions (Fig. 3*C*; upper panel; lane 4). NPM-knockdown levels (Fig. S7*A*) and Cdk1 pull-down efficiencies (Fig. 3*C*) were determined by Western blots using anti-NPM and anti-Cdk1 antibodies, respectively. Furthermore, to determine whether RASSF10-induced NPM-dependent nuclear accumulation of GADD45a alters the Cyclin-B–Cdk1 kinase complex formation, we performed the coimmunoprecipitation assay with AGS cell lysates containing RASSF10 using indicated antibodies. Results in Fig. S7*B* clearly reveal that Cdk1 forms a complex with GADD45a (middle panel; lane 2) more efficiently than with cyclin-B (upper panel; lane 2) in presence of RASSF10 expression. These results suggest that RASSF10-induced NPM is critical to inhibit the complex formation between cyclin-B and Cdk1 by promoting the nuclear transport of GADD45a. To further define the NPM dependency on RASSF10 function, status of cell proliferation was determined in AGS cells under NPM-depleted conditions with or without RASSF10 expression. Results from the MTT assay showed that RASSF10 failed to control cell proliferation under NPM-knockdown conditions (Fig. 3*D*). In support of this, results from the flow cytometry analysis clearly indicated that RASSF10 failed to induce G2/M arrest with NPM knockdown (Fig. 3*E*). Expression of RASSF10 and knockdown efficiency of NPM were determined by Western blot using anti-Flag and anti-NPM antibodies, respectively (Fig. S7*C*). It is worth to mention that RASSF10 levels were reduced or undetectable under NPM-depleted conditions (Fig. S7*C*; lane 4). These experiments were repeated multiple times, and similar results were observed. Collectively, these data provided evidence that RASSF10 inhibits Cdk1–cyclin-B kinase complex formation and induces mitotic arrest during cell division cycle in NPM-dependent manner.

To gain further insights on the role of GADD45a on RASSF10-mediated mitotic arrest, we determined the status of cell proliferation and cell cycle profiles in AGS cells with RASSF10 expression under GADD45a knockdown conditions. Results clearly suggest that RASSF10 failed to induce G2/M arrest (Fig. S8*A*) and inhibit cell proliferation (Fig. S8*B*) under GADD45a knockdown condition. Expression of RASSF10 and the efficiency of GADD45a knockdown were confirmed by Western blot analysis using indicated antibodies (Fig. S8*C*). Taken together, these results provide evidence that NPM-mediated nuclear translocation of GADD45a is critical for RASSF10 to induce mitotic arrest during cell cycle.

### NPM promotes RASSF10 stabilization by deregulating the expression of E3 ligase RNF2

**Figure 4:**
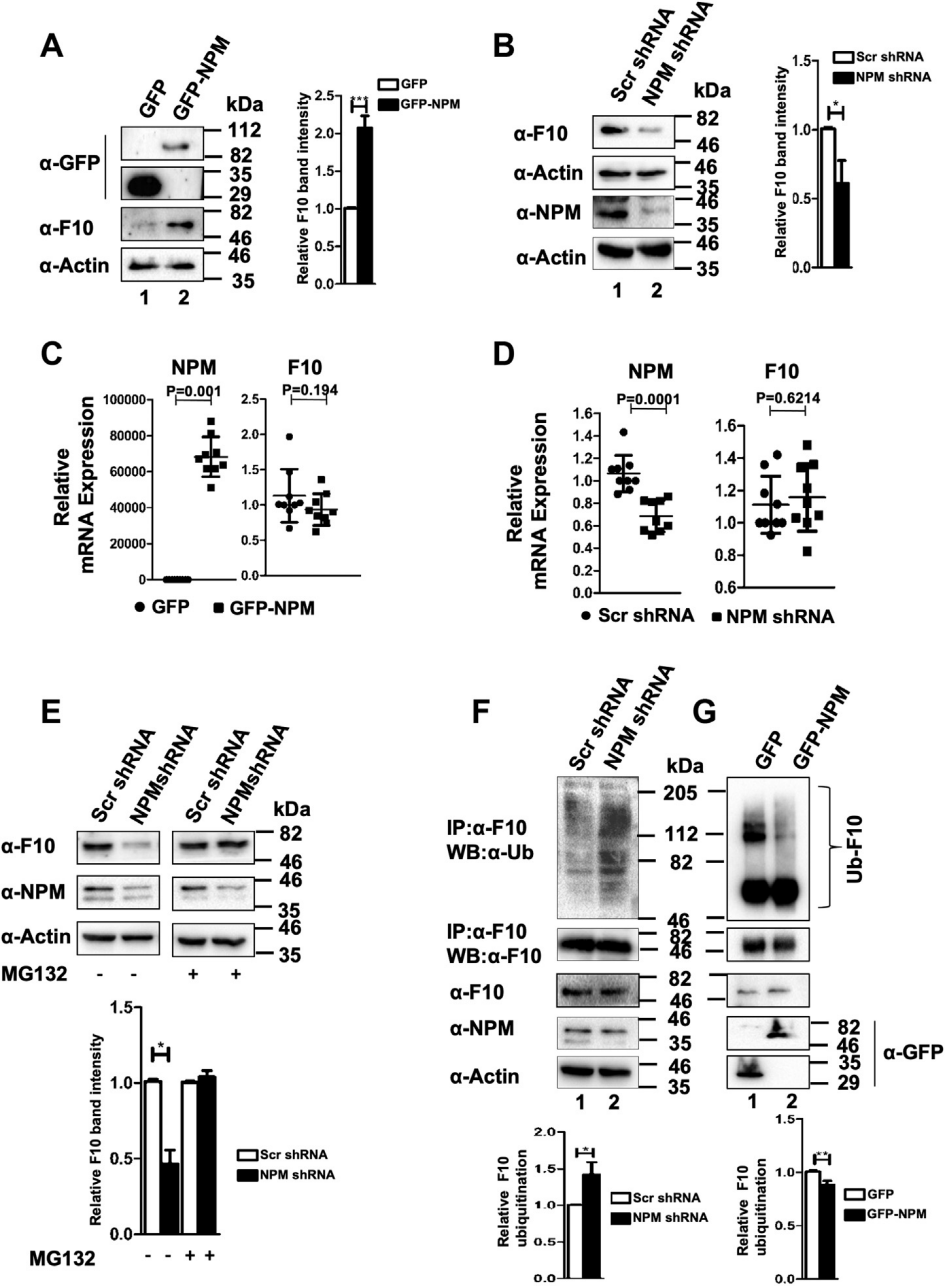
**NPM promotes RASSF10 stabilization.***A*, NPM promotes RASSF10 protein accumulation in cells. *B*, knockdown of NPM resulted in reduction of RASSF10 protein levels in AGS cells. Ectopic expression (*C*) or knockdown (*D*) of NPM resulted in no significant change of RASSF10 transcript levels. *E*, RASSF10 protein levels were stabilized under NPM-knockdown conditions in presence of proteosome inhibitor MG132. *F*, knockdown of NPM resulted in increased polyubiquitination of RASSF10 in AGS cells. *G*, ectopic expression of NPM suppressed the polyubiquitination status of RASSF10. The densitometry analysis of Western blots was carried out by normalizing the expression level of endogenous proteins to β-actin, as the loading control (n = 3, and data are expressed as the mean ± SD). F10, RASSF10; NPM, nucleophosmin; Ub, ubiquitin. (∗*p* < 0.05; ∗∗*p* < 0.01).

Data from the present study indicate that RASSF10 protein levels were diminished or undetectable in cells with NPM knockdown (Figs. S5*B*, S6*D* and S7, *A* and *C*) and suggest the possibility of feedback loop between RASSF10 and NPM, which may be critical to maintain cellular levels of RASSF10 protein. To confirm this, we checked the mRNA and protein levels of RASSF10 with perturbations of NPM levels in AGS cells. Results indicate that NPM upregulates RASSF10 protein levels (Fig. 4*A*; lane 2), and the reverse was observed with NPM-depleted conditions (Fig. 4*B*; lane 2) despite no change in RASSF10 mRNA levels (Fig. 4, *C* and *D*) under same conditions. Ectopic expression and knockdown efficiencies of NPM were determined by Western blot analysis using anti-GFP (Fig. 4*A*) and anti-NPM antibodies (Fig. 4*B*), respectively. Together, these results suggest that NPM may play a critical role in RASSF10 protein stabilization. Furthermore, the steady state levels of RASSF10 were determined in the presence or absence of NPM expression by cycloheximide (CHX) chase assay. Results in Fig. S9*A* indicate that RASSF10 protein levels were unchanged till 120 min after CHX chase with NPM expression as compared with GFP-expressed cells (RASSF10 levels were undetectable after 30 min of chase). In support of this, RASSF10 protein levels were destabilized faster and undetectable after 20 min of CHX chase under NPM knockdown conditions (Fig. S9*B*). These results suggest that RASSF10 protein levels in cells might be regulated post-transnationally by NPM.

**Figure 5:**
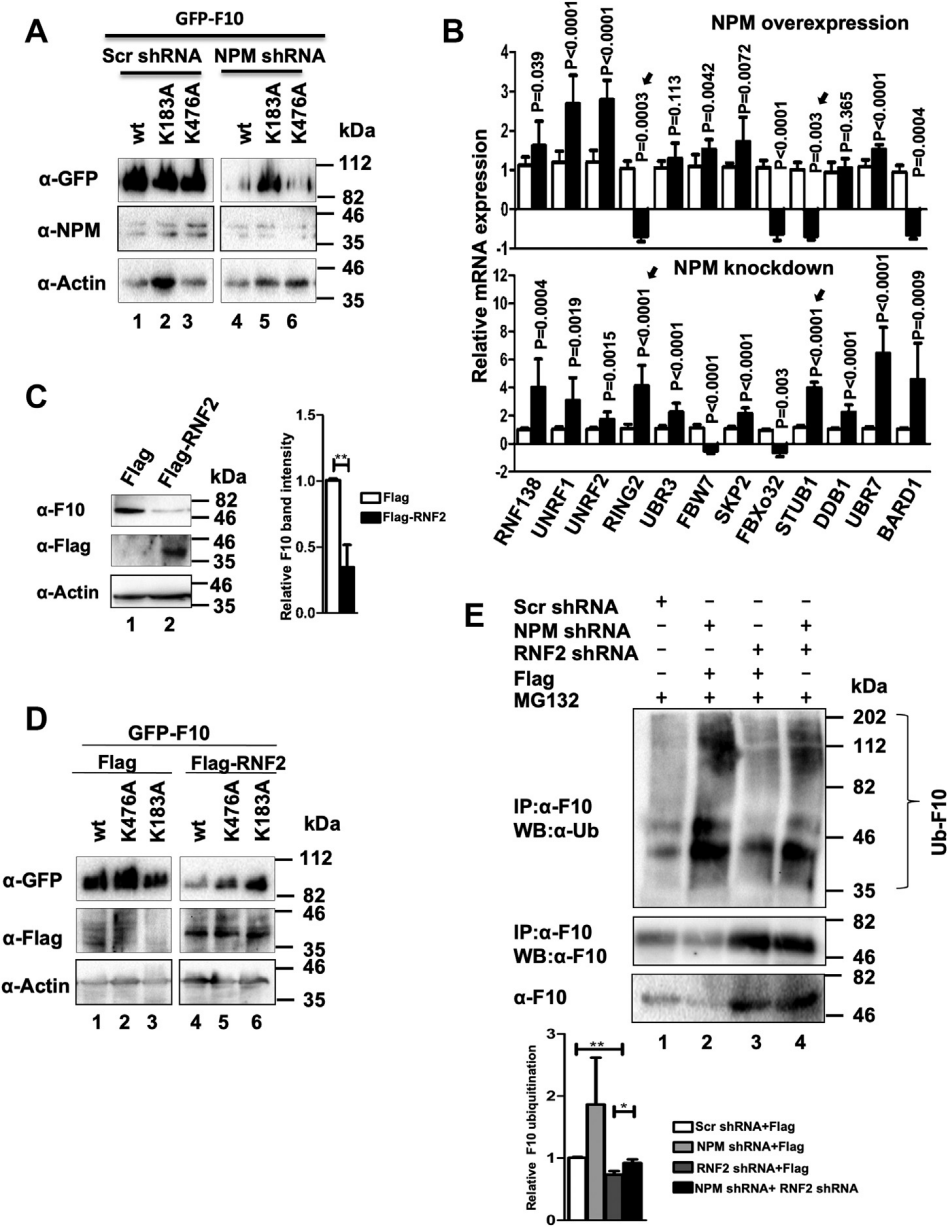
**RASSF10 is a substrate for E3 ligase RNF2, and NPM deregulates the expression of RNF2 to stabilize RASSF10 protein levels.***A*, replacement of lysine 183 not 476 stabilizes RASSF10 variant under NPM-knockdown conditions. Efficiency of NPM knockdown and the expression levels of WT and indicated mutants of RASSF10 were analyzed by Western blot using indicated antibodies. *B*, expression profile of a panel of E3 ligases was determined by RT-qPCR analysis under NPM-expression or NPM-knockdown conditions. Arrows indicate genes that are selected for subsequent experiments. *C*, ectopic expression of RNF2 resulted in significant reduction of RASSF10 protein levels. *D*, replacement of lysine 183 resulted in stabilization of mutant RASSF10 protein levels in AGS cells in presence of RNF2 expression. *E*, the knockdown of RNF2 prevents polyubiquitination of RASSF10 protein. The status of RASSF10 polyubiquitination was determined by ubiquitination assay followed by Western blot analysis using indicated antibodies. The densitometry analysis of Western blots was carried out by normalizing the expression level of endogenous proteins to β-actin, as the loading control (n = 3, and data are expressed as the mean ± SD). F10, RASSF10; NPM, nucleophosmin; Ub, ubiquitin. (∗*p* < 0.05; ∗∗*p* < 0.01).

It is well documented that proteasome-mediated degradation is one of the most important post-translational regulation of protein levels in cells (22). Data from the current investigation prompted us to test whether RASSF10 undergoes faster ubiquitination followed by proteasome-mediated degradation under NPM-knockdown condition. Results in Figure 4*E* clearly indicate that knockdown of NPM significantly reduced the levels of RASSF10 protein, whereas the proteasome inhibitor MG132 treatment stabilized RASSF10 protein under the same condition. These results suggest that NPM stabilizes RASSF10 protein levels by regulating the proteasome degradation pathway. It is well known that attachment of ubiquitin moieties to the lysine residues in a protein initiates the proteasome-mediated degradation (23). To understand the mechanism, the ubiquitination status of RASSF10 was first determined under NPM-knockdown conditions. Results in Figure 4*F* indicate that an increased RASSF10 ubiquitination was observed with NPM knockdown compared with scrambled shRNA–transfected cells (lane 2). In contrast, the reverse was observed with ectopic expression of NPM (Fig. 4*G*; lane 2). Bioinformatics analysis with RASSF10 amino acid sequences identified lysine residues at positions 183 and 476 as potential ubiquitination sites (Fig. S10*A*). To confirm this possibility, variants of RASSF10 were generated by site-directed mutagenesis. WT and indicated variants of RASSF10 were ectopically expressed in AGS cells, and the protein levels were determined under NPM-knockdown conditions. Interestingly, replacement of K183 promoted mutant protein stabilization (Fig. 5*A*; lane 5) compared with WT and K476A mutant of RASSF10 (Fig. 5*A*; lanes 4 and 6) under NPM-depleted conditions. Furthermore, the ubiquitination status of WT and indicated variants of RASSF10 was determined in AGS cells. Results in Fig. S10*B* suggest that increased ubiquitination was observed with WT and K476A variant (lanes 4 and 5) as compared with K183A mutant of RASSF10 (lane 6) under NPM-knockdown conditions. These results suggest that K183 may be the potential ubiquitination site in RASSF10 for proteasome-mediated degradation and further indicate that NPM may regulate a novel E3 ligase responsible for RASSF10 polyubiquitination/destabilization.

We next checked the expression status of the panel of E3 ligases under NPM-expression or NPM-knockdown conditions in AGS cells. Interestingly, results from RT-qPCR analysis indicate that the expression of transcripts of E3 ligases STUB1 and RING2 (RNF2) was significantly suppressed with NPM expression, and the reverse was observed under NPM-depleted conditions (Fig. 5*B*; Fig. S10*C*). Because significant inverse correlation of RNF2 expression with NPM-expression and NPM-knockdown conditions was observed after multiple repetitions of this experiment, RNF2 was selected for subsequent experiments. As expected, the ectopic expression of RNF2 resulted in reduced RASSF10 protein levels in AGS cells. (Fig. 5*C*; upper panel; lane 2). Interestingly, depletion of RNF2 by two independent shRNAs resulted in more accumulation of RASSF10 protein compared with scrambled shRNA–transfected cells (Fig. S10*D*). Knockdown levels of RNF2 were checked by RT-qPCR analysis (Fig. S11*A*). Furthermore, ectopic expression of RNF2 resulted in reduced accumulation of WT and K476A mutant of RASSF10 (Fig. 5*D*; lanes 4 and 5). However, levels of K183A mutant of RASSF10 were unaffected by RNF2 expression (Fig. 5*D*; lane 6). Correspondingly, RNF2 expression resulted in increased polyubiquitination of WT and K476A mutants of RASSF10 (Fig. S11*B*; lanes 4 and 5), and in contrast, ubiquitination levels of K183A mutant were unchanged (Fig. S11*B*; lanes 3 and 6). These data suggest that RNF2 might be responsible for ubiquitination of RASSF10 at K183. To establish the role of NPM on RNF2-mediated polyubiquitination of RASSF10, the ubiquitination status of RASSF10 was measured under RNF2- and/or NPM-knockdown conditions by coimmunoprecipitation followed by Western blot analysis. Results clearly reveal that the polyubiquitination status of RASSF10 was increased with NPM knockdown (Fig. 5*E*; lane 2). Interestingly, the same level of RASSF10 ubiquitination was noticed under RFN2-knockdown condition as well as scrambled shRNA–transfected cells (Fig. 5*E*; lanes 1 and 3), although increased RASSF10 protein pulldown was obtained under RFN2-knockdown conditions (Fig. 5*E*; middle panel; lane 3). Interestingly, this was reversed when both NPM and RNF2 were depleted (Fig. 5*E*; lane 4). The knockdown levels of RNF2 and NPM and the expression levels of RASSF10 protein were determined by Western bolt analysis using indicated antibodies (Fig. S11*C*). Collectively, these results provide evidence that RASSF10 is a potential substrate for E3 ligase RNF2 and further suggest that NPM promotes RASSF10 protein stabilization by suppressing the expression of RNF2.

### Clinical relevance of RASSF10, NPM, and RNF2 expression in gastric cancer

**Figure 6:**
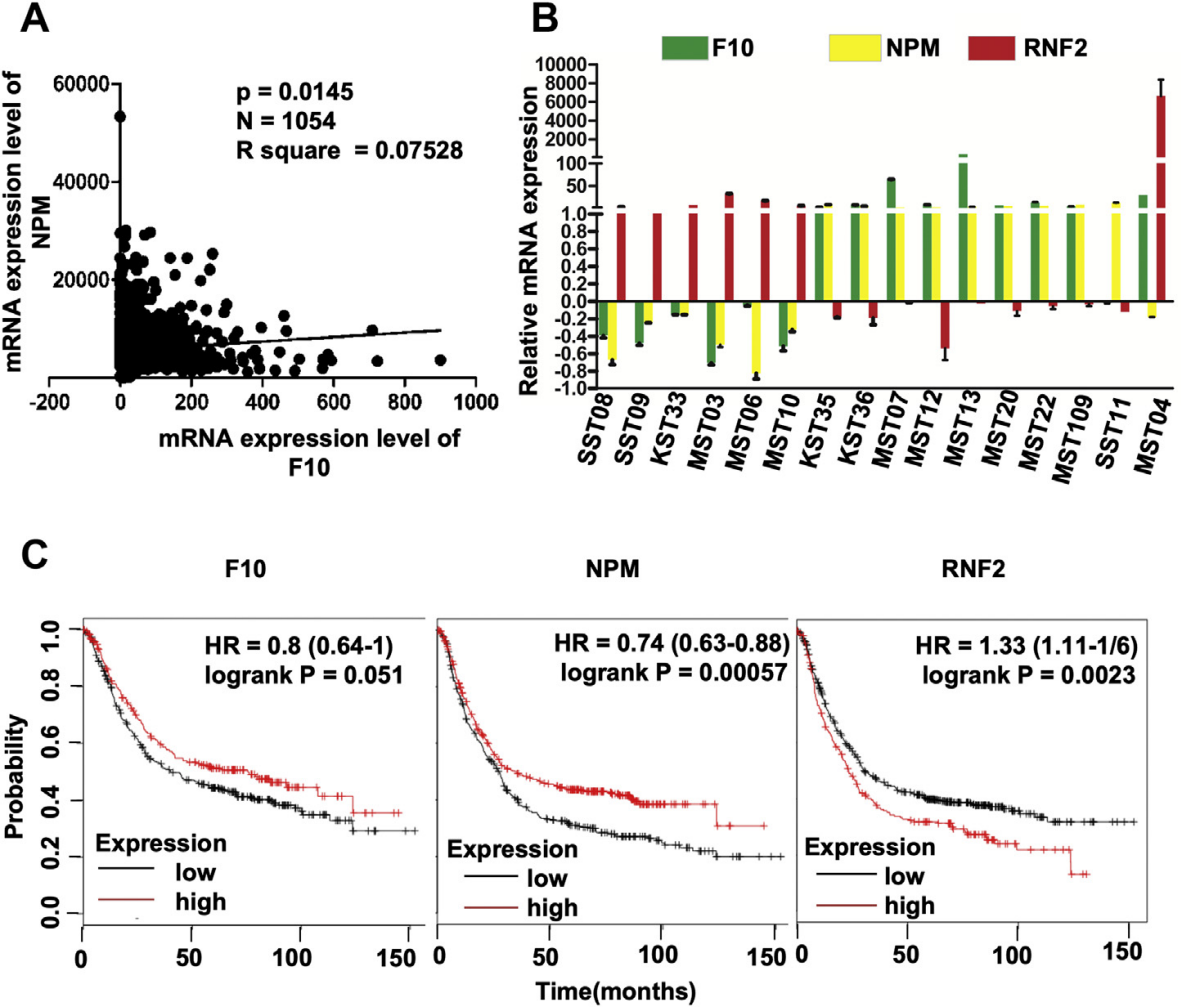
**Clinical relevance of RASSF10 and NPM expression in gastric cancer.***A*, correlation analysis for the expression frequency of RASSF10 and NPM in gastric cancer as retrieved from the BioXpress database. *B*, RASSF10, NPM, and RNF2 transcript levels were measured in Indian cohorts of stomach tumor samples by RT-qPCR. *C*, the Kaplan–Meier plot analysis using TCGA database suggests that higher expression of RASSF10 and NPM positively correlated with overall survival of patients with gastric cancer, and in contrast, a high level of RNF2 expression associated with poor survival of patients with gastric cancer. F10, RASSF10; NPM, nucleophosmin.

Having elucidated that the critical role for the RASSF10/NPM positive feedback loop on control of cell proliferation, we next checked the clinical relevance of this regulation in gastric cancer. To this end, the expression profile of RASSF10 and NPM was analyzed from BioXpress database, which utilizes RNA-Seq V2 RSEM values from the cancer genome atlas (TCGA) datasets (24). Correlation analysis suggests a significant positive correlation of RASSF10 and NPM expression in gastric cancer samples with a Pearson correlation coefficient of 0.075 (Fig. S6*A*). This is in support of the findings of current investigation that RASSF10 is positively regulating NPM expression and NPM is stabilizing RASSF10 by inhibiting polyubiquitination and proteasome-mediated degradation. To further investigate whether the similar correlation exists in Indian gastric cancer cohorts, mRNA was extracted from 16 stomach adenocarcinoma samples with respective matched normal tissue samples and the expression profile of RASSF10, NPM, and RNF2 was analyzed. Results from RT-qPCR analysis indicate a positive correlation of RASSF10 and NPM expression in 14 of 16 samples (Fig. 6*B*). Interestingly, RNF2 expression was inversely correlated with both RASSF10 and NPM expression (Fig. 6*B*). Furthermore, the Kaplan–Meier survival plot analysis was performed to define the correlation between RASSF10, NPM, and RNF2 expression levels with overall probability of patient survival using TCGA and GEO databases. Correlation analysis clearly indicates that higher expression of RASSF10 and NPM correlates with better patient survival (Fig. 6*C*); in contrast, higher expression of RNF2 correlated with poor survival of patients with gastric cancer (Fig. 6*C*). Collectively, these observations suggest the clinical significance of RASSF10, NPM, and RNF2 expression on gastric cancer prognosis.

## Discussion

**Figure 7:**
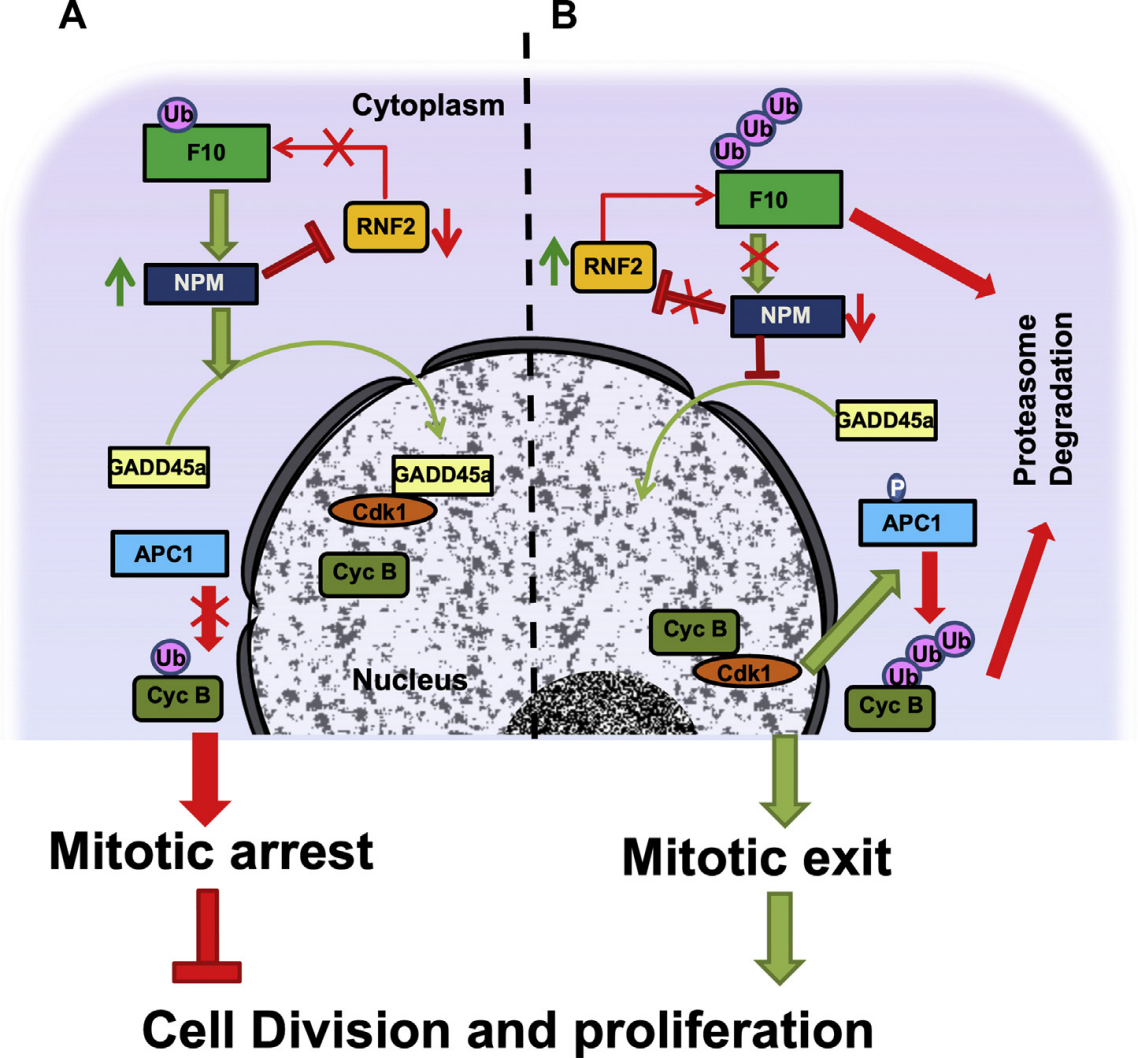
**Schematic model summarizing the critical role of NPM in RASSF10 functions during cell proliferation.***A*, in normal cells, RASSF10 upregulates the expression of NPM, which in turn promotes the nuclear translocation of GADD45a. Nuclear localized GADD45a interacts with Cdk1 and inhibits the complex formation between Cdk1 and cyclin-B, which is critical to impair APC1 phosphorylation and maintains cyclin-B levels in cells. This situation leads to mitotic arrest during cell division cycle. In addition, NPM downregulates the expression of the E3 ligase RNF2 and thereby inhibits the polyubiquitination and degradation of RASSF10. *B*, in cancer cells, suppression of RASSF10 expression due to promotor hypermethylation resulted in low levels of NPM expression. Increased expression of RNF2 under this situation may promote polyubiquitination followed by proteosome-mediated degradation of RASSF10. Accumulation of GADD45a in the cytoplasm in the absence of RASSF10 promotes cyclin-B–Cdk1 kinase complex formation, which resulted in phosphorylation of APC1. Activated APC1 induces faster polyubiquitination/degradation of cyclin-B, which resulted in mitotic exit and increased cell proliferation. APC1, anaphase-promoting complex 1; Cyc B, cyclin-B; F10, RASSF10; GADD45a, growth arrest and DNA damage–inducible alpha; NPM, nucleophosmin.

RASSF10, a member of the N-terminal RASSF, is shown to regulate cell division cycle, apoptosis, and homeostasis in cells. Downregulation of RASSF10 expression in wide range of cancers due to promoter hypermethylation (3, 5, 7, 9) resulted in uncontrolled cell proliferation, but the mechanism(s) remains poorly understood. For the first time, the present study demonstrates that RASSF10 induces mitotic arrest to control cell cycle progression by inhibiting Cdk1–cyclin-B kinase complex formation. Furthermore, using proteomics approach, NPM was identified as a novel functional target for RASSF10. RASSF10 promotes the nuclear accumulation of GADD45a to induce mitotic arrest in NPM-dependent manner, supporting the notion that NPM is critical for RASSF10 function. Interestingly, RASS10 is a substrate for E3 ligase, RNF2, and it is shown that NPM regulates the expression of RNF2 to stabilize RASSF10 protein levels in cells. Finally, the expression of RASSF10 and NPM positively correlated with the survival of patients with gastric cancer, and the reverse was observed with RNF2 expression, which suggests their association with gastric cancer progression. Collectively, our data suggest that RASS10/NPM/RNF2 feedback signaling cascade (Fig. 7) may act as a potential drug target to identify novel therapeutics for gastric cancers.

NPM, a nucleocytoplasmic shuttling protein, has been implicated in a number of pathways including mRNA transport, chromatin remodeling, apoptosis, ribosomal biogenesis, transport of preribosomal particles, modulation of protein nuclear localization, and genome stability (25). Using the proteomic approach, NPM was identified as a functional target for RASSF10 to regulate cell division cycle (Fig. 3). It is known that NPM modulates p53/GADD45a signaling to regulate cell cycle progression (17). In 60% of primary acute myeloid leukemia, the C-terminal mutant NPM was localized to the cytoplasmic compartments and promotes faster cell proliferation (26, 27). Together, these data suggest that nuclear localization of NPM is critical for tumor-suppressive function. This is in accordance with results from the present study that RASSF10-dependent upregulated NPM translocates GADD45a to a nuclear compartment to induce mitotic arrest during cell cycle. NPM was shown to regulate DNA repair as well as the polyubiquitination of ATF5 in hepatocellular carcinoma cells (28) and reported to be a pro-oncogenic and antioncogenic factor in context-dependent manner (29). Consistent with this, NPM was found to alter the ubiquitination and proteosome-dependent degradation of RASSF10 by suppressing the expression of E3 ligase RNF2, which is critical to maintain RASSF10 levels in cells to induce mitotic arrest. Collectively, these data provide evidence that NPM dependency is critical for RASSF10 to control cell proliferation. The positive correlation of RASSF10 and NPM expression with better patient survival further suggests the positive feedback axis between RASS10 and NPM plays a critical role in controlling gastric cancer progression.

GADD45a is one of the p53-regulated genes, and being a stress sensor of the cell, its expression is induced under genotoxic and nongenotoxic stress conditions (30). Recent report suggests that GADD45a induces G_2_/M arrest and controls cell division cycle (31). GADD45a was found to be localized in the cytoplasm of oral squamosal cell carcinoma cells in contrast to its nuclear localization in normal cells, suggesting that nuclear localization of GADD45a is important for cell cycle regulation (32). In support of these findings, the results from the current investigation demonstrate that the knockdown of *GADD45a* by shRNA exhibited a defect in RASSF10-dependent G_2_/M arrest and further showed that RASSF10 mediated nuclear localization of GADD45a is critical to induce mitotic arrest. Interestingly, depletion of NPM with shRNA resulted in cytoplasmic localization of GADD45a despite RASSF10 expression in cells. It is worth to mention that results from the current investigation together with the existing literature demonstrate that RASSF10 (Fig. S4*C*) NPM (Fig. S4*C*; (19, 33, 34)), and Cdk1–cyclin-B complex (35, 36, 37) are localized to the nuclear compartment. Cdk1–cyclin-B kinase complex formation occurs efficiently in absence of GADD45a in the nuclear compartment and activates APC1 followed by cyclin-B ubiquitination and proteasome-mediated degradation, which is critical for inducing mitotic exit and increased cell proliferation. On the contrary, the nonavailability of the CdK1–cyclin-B kinase complex leads to inactivation of APC1 and resulted in maintenance of stable cyclin-B levels, which precludes the cells from mitotic exit to control cell proliferation. It is worth mentioning that GADD45a localized to the nuclear compartments in presence of RASSF10 expression despite lack of nuclear localization signal. Existing literature suggests that certain proteins are translocated to the nuclear compartment by nuclear localization signal–independent and importin-independent pathways through piggy-back mechanism (38). Furthermore, NPM has been found to play an important role in transporting some proteins into the nucleus to regulate cell proliferation (39). Most interestingly, as discussed above, RASSF10 promotes GADD45a nuclear localization in NPM-dependent manner and showed that GADD45a nuclear localization is critical for RASSF10-induced G_2_/M arrest during cell cycle. Together, these results provide evidence that NPM is indispensable for RASSF10-dependent nuclear localization of GADD45a to induce efficient mitotic arrest during cell division cycle. Collectively, the present investigation provides evidence that the cross-talks between NPM, GADD45a, and other cell cycle regulators coordinate the function of RASSF10 in response to genotoxic stress.

RNF2 is an important member of polycomb group of proteins, a catalytic subunit of the PRC1 complex and a key regulator of H2A monoubiquitination during development (40). In addition, RNF2 also known to polyubiquitinate many cellular proteins including TP53 and regulate cell division, apoptosis, cell proliferation, and autophagy (41, 42, 43). Recently, it has been reported that RNF2 destabilizes p53 in different cancer types (44), providing a possible mechanism of how RNF2 functions as an oncogene. In support of this, the present study showed that RNF2 promotes the polyubiquitination and proteasome-mediated degradation of RASSF10. Interestingly, the expression of RNF2 as well as knockdown of NPM in gastric cancer cells enhance RASSF10 ubiquitination and destabilization, and the reverse was noticed with depletion of endogenous *RNF2* by shRNA or ectopic expression of NPM. It has been reported that RNF2 was expressed in very low levels in normal tissues but was highly expressed in certain cancer types (45) and considered to be a prognostic biomarker and potential therapeutic target for various cancers. Interestingly, higher expression of RNF2 induces cisplatin resistance in ovarian cancers, and its knockdown enhanced the radiosensitivity of lung cancer cell lines, suggesting its role in promoting tumorigenesis (46, 47). In support of this, the survival plot analysis using data from TCGA database showed a negative correlation of RNF2 expression with poor patient survival, which suggests its role in gastric cancer progression. Furthermore, an inverse correlation was observed between RNF2 and RASSF10 expression in Indian gastric cancer cohorts. Recent report suggests that knockdown of *RNF2* arrests cells at the G2/M phase (48). Consistent with this, present investigation demonstrates that the knockdown of *RNF2* promotes RASSF10 expression and induces efficient mitotic arrest during cell cycle. Taken together, results from the current investigation provided evidence that the observed NPM-dependent downregulation of RNF2 may be essential for maintaining RASSF10 levels to induce G2/M arrest for controlling cell proliferation.

Most of the chemotherapeutic drugs suppress tumor growth by inducing prolonged cell cycle arrest followed by apoptosis (49, 50, 51). Microtubule inhibitors, a class of chemotherapeutic drugs, specifically arrest cells at the mitotic phase of the cell division cycle (52). It is well known that maintenance of stable cyclin-B levels is critical to induce mitotic arrest (53). Results from the present study for the first time provided evidence that RASSF10 promotes the nuclear translocation of GADD45a in NPM-dependent manner, which is critical to maintain stable cyclin-B levels for efficient mitotic arrest. In addition, the present investigation showed that RNF2 is responsible for polyubiquitination and proteasome-mediated degradation of RASSF10. Collectively, these data elucidate that RASSF10 regulates cell proliferation by modulating a novel NPM/GADD45a/RNF2 signaling axis (Fig. 7). Furthermore, studies on NPM-mediated suppression of RNF2 expression and role of RASSF10 on cell cycle regulation in presence of chemotherapeutic drugs might help in designing novel cancer treatment strategies. Taken together, our findings provide strong evidence that NPM, E3 ligase RNF2, and GADD45a directly and functionally control powerful RAS effector networks that are vital in multiple cancer processes. In conclusion, RASSF10 is an important downstream target of RNF2 and the RASSF10/NPM/GADD45a/RNF2 feedback cascade may be used as a new biomarker for diagnosis and novel drug target for the therapy of gastric cancer.

## Experimental procedures

### Plasmid construction

RASSF10 (NM_001080521) was amplified from human peripheral blood mononuclear cell cDNA using appropriate primers (Table S3) and cloned between EcoRI and XhoI sites of the pCDNA3.1 vector as GFP or Flag fusions. RASSF10 variants were generated using GFP-RASSF10 plasmid as the template with appropriate primers (Table S3). RNF2 was amplified from human peripheral blood mononuclear cell cDNA using appropriate primers (Table S3) and cloned between KpnI and XhoI sites of pCDNA3.1-Flag plasmid. NPM expression plasmid was purchased from Addgene. For knockdown studies, shRNAs for GADD45a (TRCN0000062350), RASSF10 (TRCN0000255400), RNF2 (TRCN0000033697), TP^53^ (TRCN0000003753), NPM (TRCN0000062270), and control shRNA (SHCO16) were purchased from Sigma Aldrich. Target sequences of shRNAs are detailed in Table S3. All expression clones were sequenced to verify the integrity.

### Antibodies, chemicals, and reagents

Antibodies and other reagents used in this study are detailed in Table S4.

### Patient samples

The study was approved by the Institutional Ethical Board (Ref No: IEC/2016/01/SM-6/16) of Indian Institute of Technology Madras, Chennai, India, in accordance with the Declaration of Helsinki ethical guidelines. All methods were performed in accordance with the regulations approved by the committee. Informed consent was obtained from patients before tissue sample collection. All the tumor samples and the adjacent normal samples used in this study were obtained from National Cancer Tissue Biobank, Indian Institute of Technology Madras, Chennai. Tumor and normal tissue samples were snap-frozen and stored in liquid nitrogen until used for RNA extraction. Details of all the tissue samples are described in Table S5.

### Cell culture, transfection, immunoprecipitation, and Western blot analysis

AGS, HCT116^p53−/−^, and HEK293T cells were cultured in Dulbecco's modified Eagle's medium (DMEM) (Thermo Fisher Scientific Inc) with 10% fetal bovine serum and 1% Antibiotic-Antimycotic (Thermo Fisher Scientific Inc). Identity of the cell lines was verified through sequencing, for identification of genetic mutations reported by the American Type Culture Collection. Cell lines were periodically tested for *Mycoplasma* contaminations through PCR amplification of *Mycoplasma*-specific 16S rRNA sequences according to manufacturer's instructions (Sigma-Aldrich). Cells were grown to 60% confluency and transfected with polyethyleneimine (PEI) as described previously (54). Transfected cells were lysed using 1× cell lysis buffer (25 mM Tris HCl, pH7.4, 150 mM KCl, 1 mM Na_2_EDTA, 1 mM EGTA, 1% Triton X-100, 2.5 mM sodium pyrophosphate, 1 mM β-glycerophosphate, 0.4 mM PMSF, 1 mM NaF, 1 mM Na_3_VO_4_, and 1 μg/ml each of aprotinin, leupeptin, and pepstatin). Proteins were separated on SDS-PAGE, transferred onto PVDF membrane (PerkinElmer), and probed with indicated antibodies. HRP-conjugated specific secondary antibodies (Bio-Rad Laboratories) were incubated with protein–antibody complexes and detected using the Enhanced Chemiluminescence Prime detection system (GE healthcare).

### MTT assay and cell cycle analysis

GFP-RASSF10 or Flag-RASSF10 was transiently cotransfected into the monolayer culture of AGS cells with shRNAs specific to NPM or RASSF10 or GADD45a or RNF2 alone or in combination using PEI as described above. The MTT assay or cell cycle analysis was performed 48 or 72 h after transfection as described previously (55, 56). GFP or Flag expression plasmids were used as controls.

### BrdU incorporation assay

For BrdU incorporation assay, AGS cells were transfected with indicated plasmids. Forty-eight hours or 72 h after transfection, cells were pulse-labeled with BrdU for 5 h. Labeled cells were fixed, permeabilized, and stained with anti-BrdU antibodies conjugated with allophycocyanin, followed by total DNA staining with 7-AAD as per manufacturer’s instructions (BD Biosciences). The amount of BrdU incorporation was analyzed by flow cytometry (FACSCanto II, BD Biosciences) and the data were analyzed using FACSDIVA software (BD Biosciences).

### Cell counting

AGS cells (2 × 10^5^) were seeded into each well of a 12-well plate and transiently transfected with GFP-RASSF10 or RASSF10 shRNAs. Cells were trypsinized at different time intervals, and viable cell numbers were counted using trypan blue dye. GFP and scrambled shRNA plasmids were used as controls.

### Live cell imaging

AGS cells were transfected with GFP or GFP-RASSF10 expression vector. Twenty-four hours after transfection, cells were incubated with thymidine (10 mM) for 16 h, followed by three washes with PBS, and allowed to grow in complete DMEM for additional 8 h. Cells were again treated with thymidine (10 mM) for 8 h followed by three washes with PBS and allowed to grow in complete DMEM (57). The cells were then imaged for 24 h continuously in a humidified chamber attached to an LSM 880 confocal microscope (Carl Zeiss) with 5% CO_2_, and image acquisition was performed using Zen 2009 software (Carl Zeiss)

### RT-qPCR

Total RNA from cells transfected with different plasmids was extracted using the TRIzol reagent according to manufacturer's instructions (TAKARA). RNA was converted to cDNA using reverse transcriptase according to the manufacturer’s instructions (TAKARA). RT-qPCR analysis was performed as described elsewhere (58). Expression levels of various genes relative to beta-actin were analyzed using ΔCτ values according to the manufacturer’s directions (Eppendorf). The primers used for the RT-qPCR analyses are listed in Table S3.

### Nucleocytoplasmic extraction

AGS or HCT116^p53−/−^ cells were cotransfected with Flag-RASSF10 plasmid alone or in combination with shRNA specific to NPM. Forty-eight hours after transfection, cells were washed with PBS and lysed with the nucleocytoplasmic fractionation buffer according to the manufacturer’s instructions (Thermo Fisher Scientific Inc). The lysate equivalent to 30 μg of protein was resolved on SDS-12% PAGE followed by Western blot analysis using indicated antibodies as described above.

### Immunofluorescence

AGS cells grown on cover slips (Thermo Fisher Scientific Inc) were transfected with GFP or GFP-RASSF10 expression plasmids alone or in combination with scrambled shRNAs specific to NPM using PEI. For determining the subcellular distribution of proteins, transfected cells were fixed using 3% (w/v) paraformaldehyde and permeabilized with Triton X-100. Endogenous proteins were stained with specific antibodies for 1 h at room temperature or overnight at 4 °C. Hoechst 33342 was used to stain the nuclei at a final concentration of 1 mg/ml. Samples were then viewed with an LSM 880 confocal microscope (Carl Zeiss), and image acquisition was performed using Zen 2009 software (Carl Zeiss).

### Two-dimensional gel electrophoresis and nLC-MS/MS analysis

HEK293T cells were transfected with GFP or GFP-RASSF10 plasmids. Forty-eight hours after transfection, cells were lysed in a buffer containing 7 M urea, 2 M thiourea, 4% CHAPS, 20 mM PMSF, and 20 mM DTT, and protein estimation was performed using the Bradford method before being aliquoted and stored at −80 °C for further analysis. Two-dimensional gel electrophoresis was performed as described elsewhere (58) with the following modifications. IPG strips (11 and 7 cm) of pH 3 to 11 and 4 to 7 (GE Healthcare), respectively, were used for first dimension.

Separated proteins were stained with colloidal Coomassie Brilliant Blue G-250, and the protein spots were excised manually for in-gel trypsin digestion as described previously (58), and nLC-MS/MS was performed. MS data were acquired in the positive-ion mode over mass range m/z 350 to 4000 Da using Xcalibur software (version 2.2.SP1.48) (Thermo Scientific). MS data were analyzed using Proteome Discoverer software v.1.4 (Thermo Scientific) using SEQUEST algorithm with database from UniProt as described elsewhere (58).

### Ubiquitination assay

AGS cells transiently transfected with plasmids expressing GFP-RASSF10 or shRNA specific to RASSF10, NPM, or RNF2 were treated with 20 μg/ml MG132 for 8 h after 40 h of transfection. Scrambled shRNA and GFP were used as controls. Cells were lysed with NP40 lysis buffer, and 30 μg of protein was used for Western blot analysis, or 300 μg of protein lysate was used for immunoprecipitation with anti-RASSF10 or anti-Cyclin-B antibodies followed by Western blot analysis using anti-ubiquitin antibodies.

### Gene expression analysis from TCGA

For expression and patient survival correlation analysis, expression fold change of RASSF10, NPM, and RNF2 in gastric cancer samples was retrieved from BioXpress database (http://hive.biochemistry.gwu.edu/tools/bioxpress) (59). The expression frequency was calculated from the fold change in tumor samples relative to respective normal samples. The Kaplan–Meier plot analysis for survival of the patients with gastric cancer was performed using the online Kaplan–Meier plot database (http://www.Kmplot.com) (60).

### Bioinformatics analysis for ubiquitination site prediction

Ubiquitination site prediction software tools UbPred (http://www.ubpred.org) (61) and BDM-PUB (http://www.bdmpub.biocuckoo.org) (https://www.scienceopen.com/document?vid=cdb6582a-8d17-4b0a-aae0-7044b4d6363a) were used for predicting the ubiquitination sites on RASSF10.

### Functional classification of proteins and pathway analysis

Protein ANalysis THrough Evolutionary Relationships (PANTHER) database (16) was used for functional classification of all the proteins identified to be differentially regulated by RASSF10 in two-dimensional gel electrophoresis and nLC-MS/MS analysis. Biological processes were selected in ontology for further analysis.

### Statistical analysis

GraphPad Prism 5.0 software was used for performing the statistical analysis. In all the RT-qPCR, MTT, and BrdU analysis, error bars represent the mean ± SD from three independent experiments except for the RT-qPCR analysis of RASSF10, NPM, and RNF2 levels in gastric tumor tissue samples, where the error bars represent the mean ± SEM from technical duplicates. Cell cycle analysis data are the representative of three independent experiments, and the error bars represent the mean ± SD from biological triplicates. Statistical significance was obtained by using Student’s unpaired *t* test. Western blots are the representative of three independent experiments. Densitometric analysis was performed for all the blots using ImageJ software and represented as the mean ± SD from triplicates. Statistical significance was obtained using Student’s unpaired *t* test and indicated by the *p* value (∗*p* < 0.05, ∗∗*p* < 0.01, and ∗∗∗*p* < 0.001).

## Data availability

All data generated or analyzed during this study are included in this published article and its supporting information and tables, or from the corresponding author upon request.

## Supporting information

This article contains supporting information.

## Conflict of interest

The authors declare that they have no conflicts of interest with the contents of this article.
