# Supplementary material for: Molecular basis for RASSF10/NPM/RNF2 feedback cascade–mediated regulation of gastric cancer cell proliferation
Source: J Biol Chem. 2021 Jul 3;297(2):100935. doi: 10.1016/j.jbc.2021.100935 (PMC8339327; doi:10.1016/j.jbc.2021.100935)
Supplement: Supplemental Figures S1–S11 and Tables S1–S5 [file mmc3.pdf]

Molecular basis for RASSF10/NPM/RNF2 feedback cascade-mediated regulation of gastric cancer cell proliferation

Naga Padma Lakshmi Ch, Ananthi Sivagnanam, Sebastian Raja, Sundarasamy Mahalingam*

Sundarasamy Mahalingam,
E-mail: mahalingam@iitm.ac.in

This PDF file includes:

Figures S1 to S11

Tables S1 to S5

Legends for Movies S1 and S2

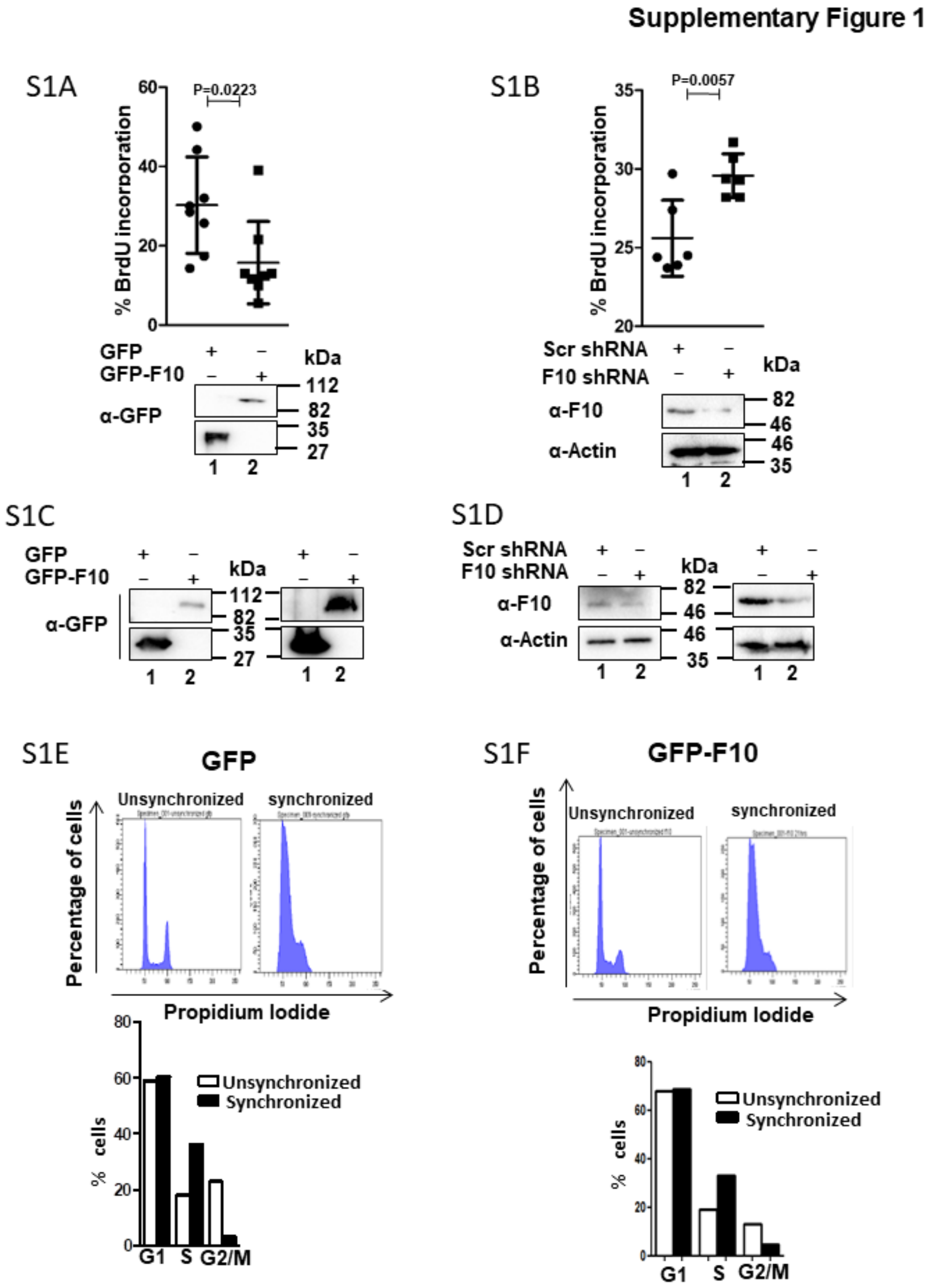

Figure S1: BrdU incorporation in AGS cells with (A) ectopic expression and (B) knockdown of RASSF10. The status of RASSF10 expression (C) and knockdown (D) for MTT and Cell counting experiments were analysed by western blot analysis using indicated antibodies. AGS cells were transiently transfected with GFP (E) and RASSF10 (F) and synchronized at G1 phase by double thymidine block before being used for cell cycle analysis. RASSF10 referred as F10.

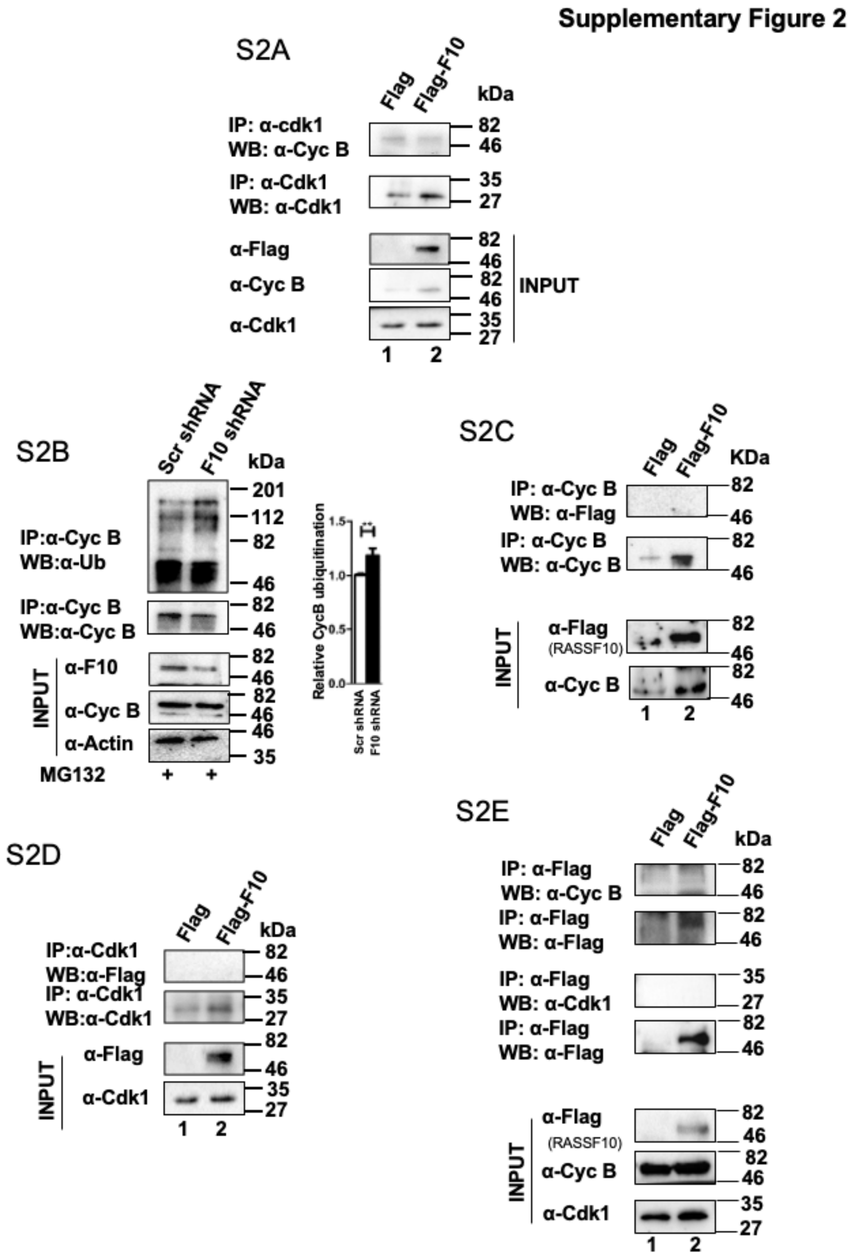

Figure S2: (A) RASSF10 expression alters the interaction between cyclin-B and Cdk1 in AGS cells. (B) Knockdown of RASSF10 promotes cyclin-B polyubiquitination. Results from the coimmunoprecipitation experiments suggest that neither cyclin-B (C) nor Cdk1 (D) interacts with RASSF10. (E) Reverse coimmunoprecipitation assay confirms that RASSF10 interacts with neither cyclin-B nor Cdk1. The densitometry analysis of western blots was carried out by normalizing the ubiquitination level of endogenous proteins to the amount of protein in pulldown as loading control (n=3 and data is expressed as mean ± SD). RASSF10 referred as F10; Cyclin-B referred as Cyc B. (*$p<0.05$; **$p<0.01$).

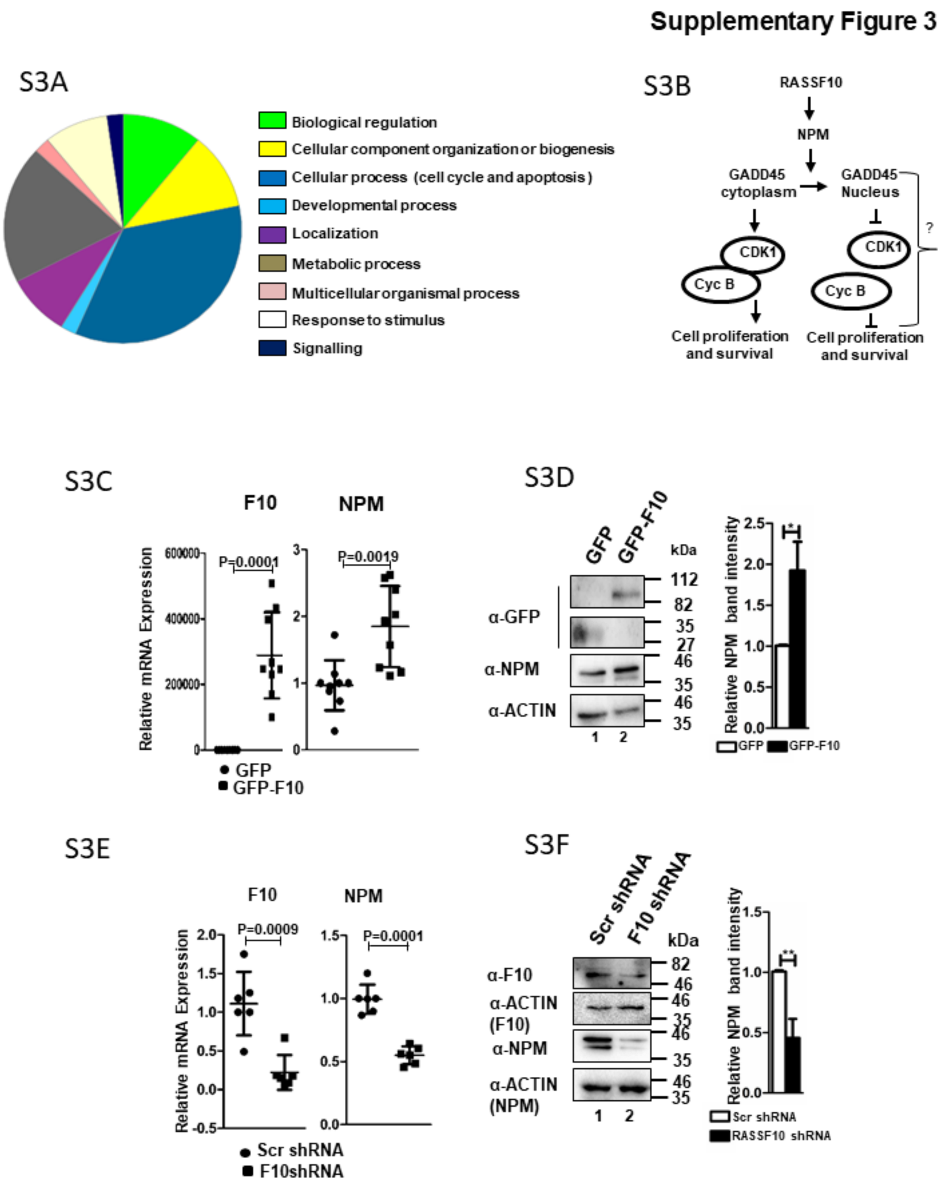

Figure S3: (A) Pathway analysis for RASSF10 deregulated proteins identified by proteomics. (B) Schematic working model on RASSF10 mediated mitotic arrest during cell cycle. RASSF10 modulates NPM levels and facilitates the nuclear translocation of GADD45a to alter the cyclin-B/Cdk1 kinase complex formation to control cell proliferation. RASSF10 upregulates the levels of NPM transcript (C) and protein (D) in AGS cells. RASSF10 knockdown resulted in reduction of NPM transcript (E) and (F) protein levels. The densitometry analysis for western blots was carried out by normalizing the expression level of endogenous proteins to β-actin, as loading control (n=3 and data is expressed as mean ± SD). RASSF10 referred as F10. (*$p<0.05$; **$p<0.01$).

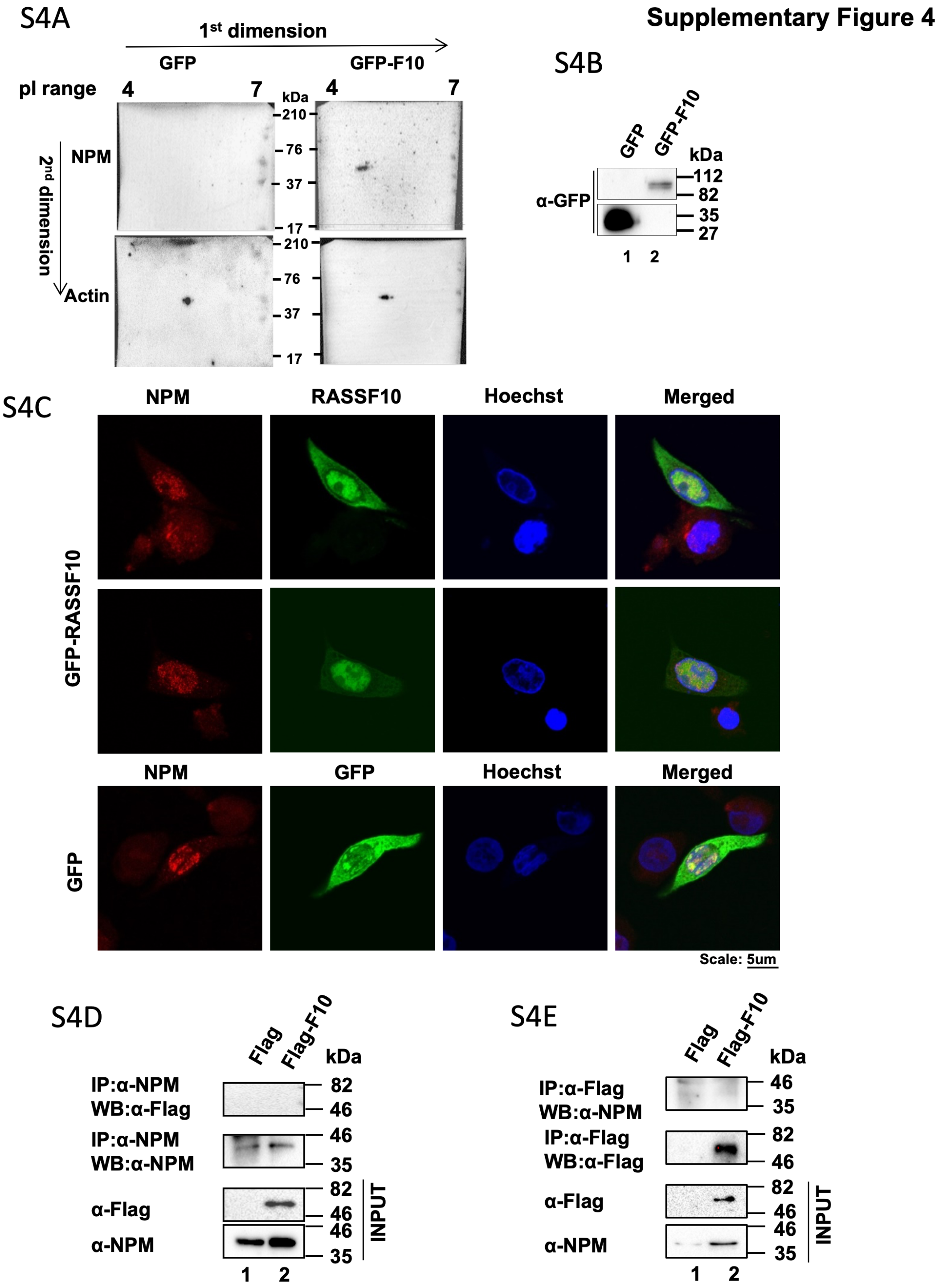

Figure S4: (A) Two-dimensional electrophoresis (2DE) followed by western analysis indicates that RASSF10 upregulates NPM protein levels in AGS cells. (B) Expression levels of RASSF10 in AGS cell lysates used for 2DE analysis. (C) RASSF10 and NPM are localized to the nucleus in contrast to the cytoplasmic localization of GFP. Results from the co-immunoprecipitation experiments with anti-NPM antibody (D) and anti-Flag antibody (E) suggest that there is no interaction between RASSF10 and NPM.

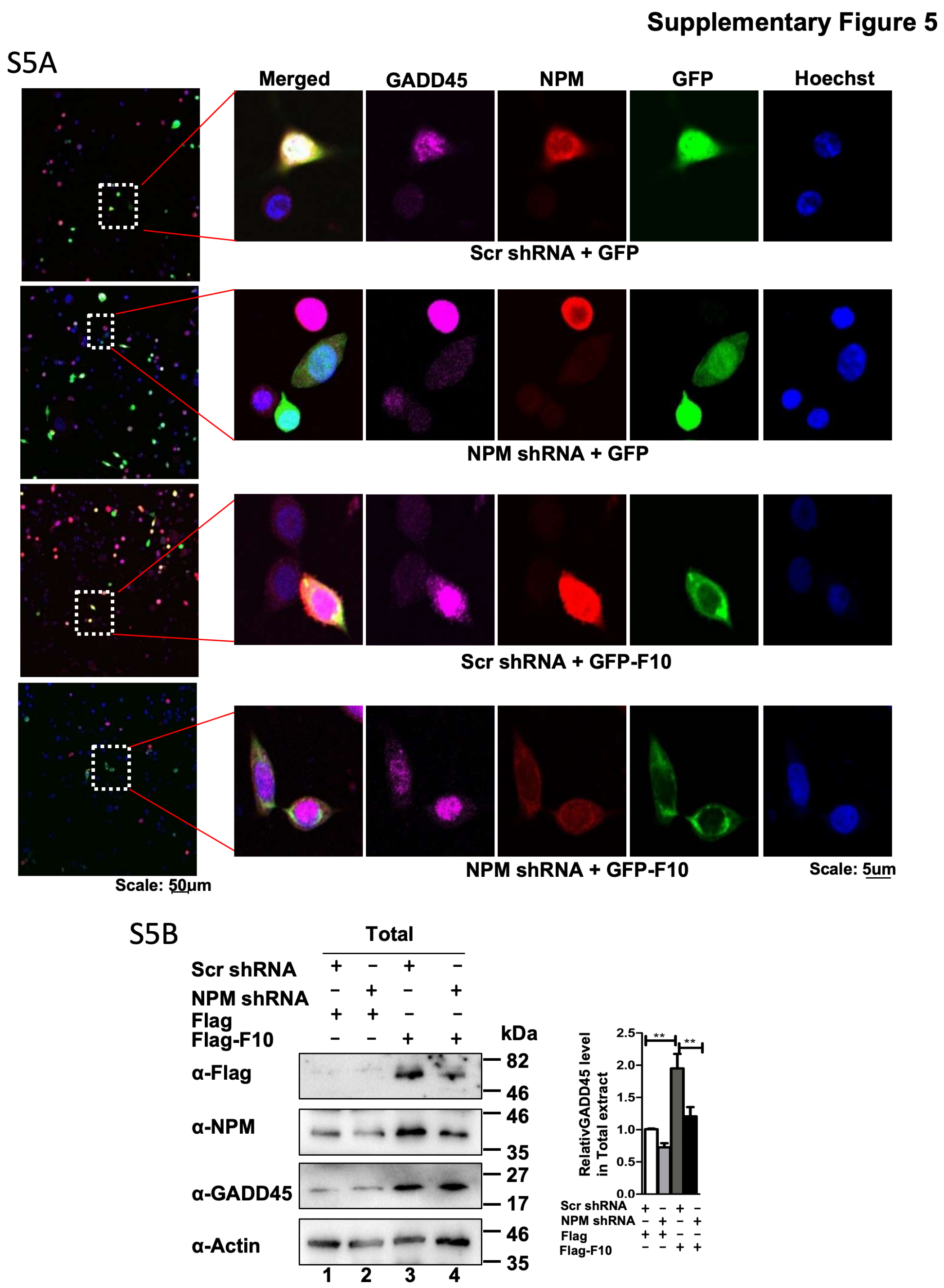

Figure S5: (A) RASSF10 facilitates the translocation of GADD45a to the nuclear compartment in NPM dependent manner in AGS cells. (B) The expression levels of RASSF10, GADD45a and the efficiency of NPM knockdown were analysed by western blot using indicated antibodies. The densitometry analysis for western blots was carried out by normalizing the expression level of endogenous proteins to β-actin, as loading control (n=3 and data is expressed as mean ± SD). RASSF10 referred as F10. (*p<0.05; **p<0.01).

Supplementary Figure 6

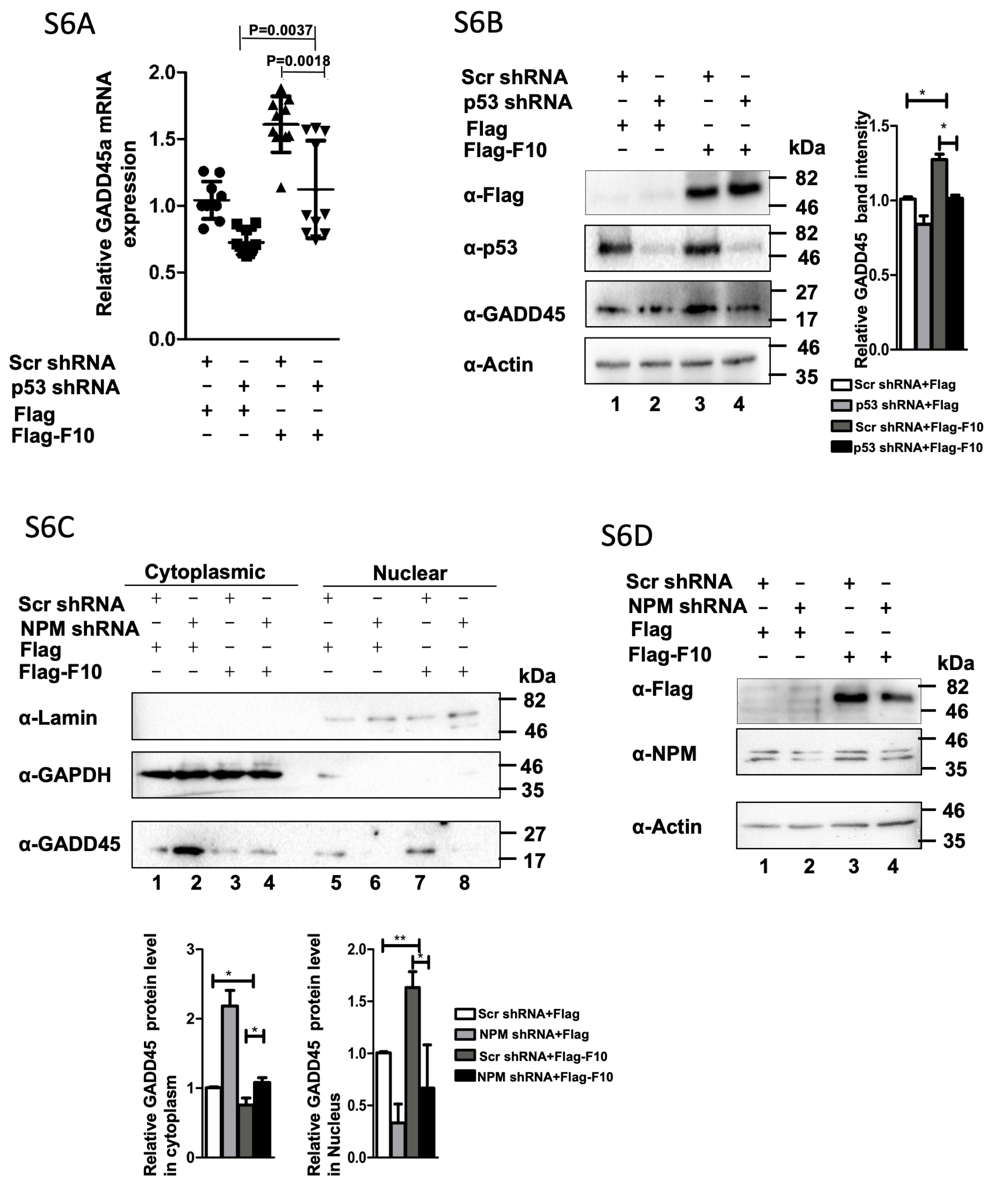

Figure S6: (A) RT-qPCR analysis indicates the dependency of p53 on RASSF10 mediated upregulation of GADD45a transcription. (B) Efficiency of p53 knockdown and the expression of RASSF10 and GADD45a were analysed by western blot using indicated antibodies. (C) RASSF10 mediated nuclear accumulation of GADD45a is NPM dependent in HCT116p53$^{-/-}$ cells. (D) Expression of RASSF10 and knockdown efficiency of NPM were determined by western blot analysis. The densitometry analysis of western blots was carried out by normalizing the expression level of endogenous proteins to β-actin, as loading control (n=3 and data is expressed as mean ± SD). RASSF10 referred as F10. (*p<0.05; **p<0.01).

Supplementary Figure 7

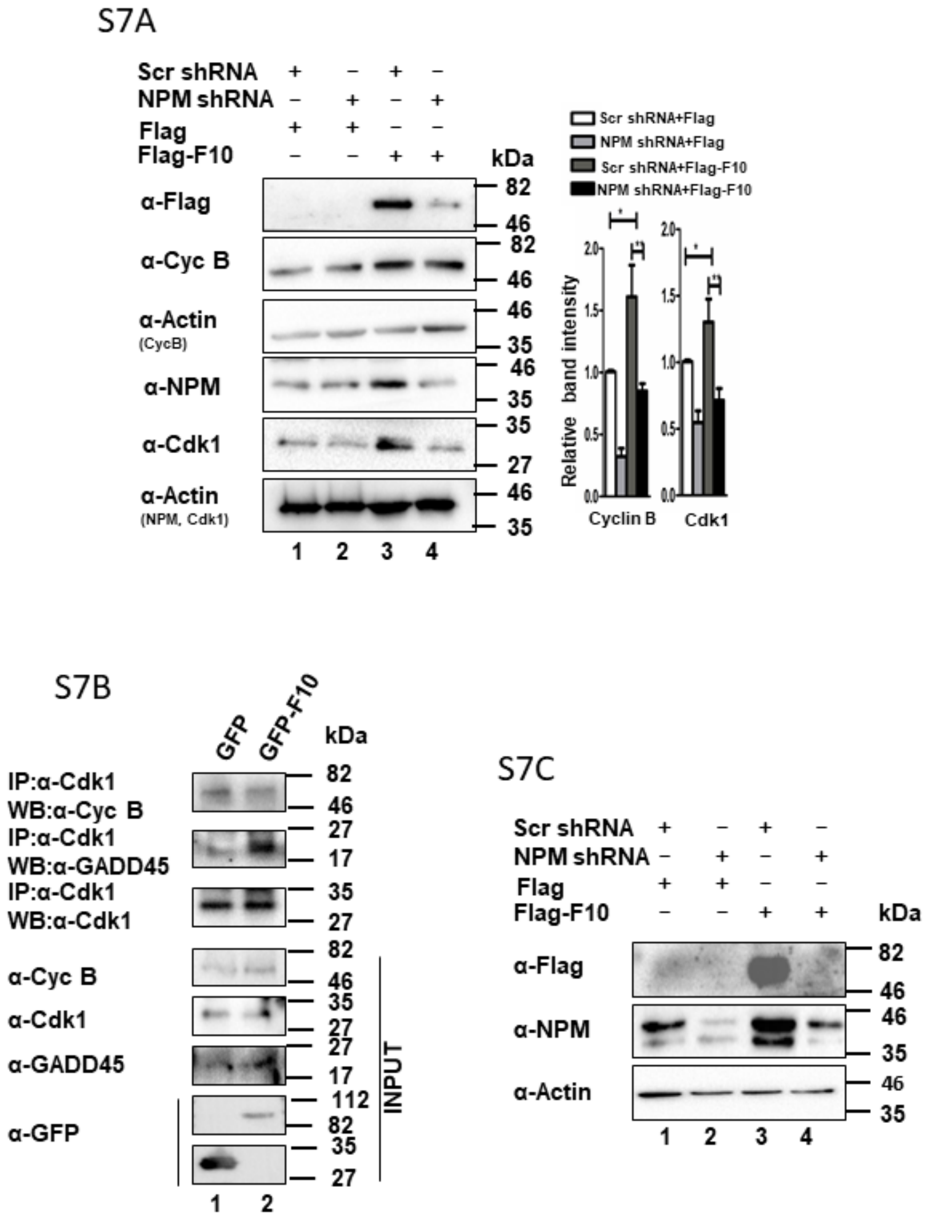

Figure S7: (A) Expression of RASSF10 promotes cyclin B and Cdk1 protein levels and this effect was reversed with the knockdown of NPM. (B) Ectopic expression of RASSF10 promoted the GADD45a association with Cdk1 and in contrast, reduced complex formation between cyclin-B and Cdk1. (C) Knockdown of NPM resulted in destabilization of RASSF10 in cells. Western blot analysis was performed to determine the protein levels of RASSF10 and NPM using indicated antibodies. (*p<0.05; **p<0.01).

Supplementary Figure 8

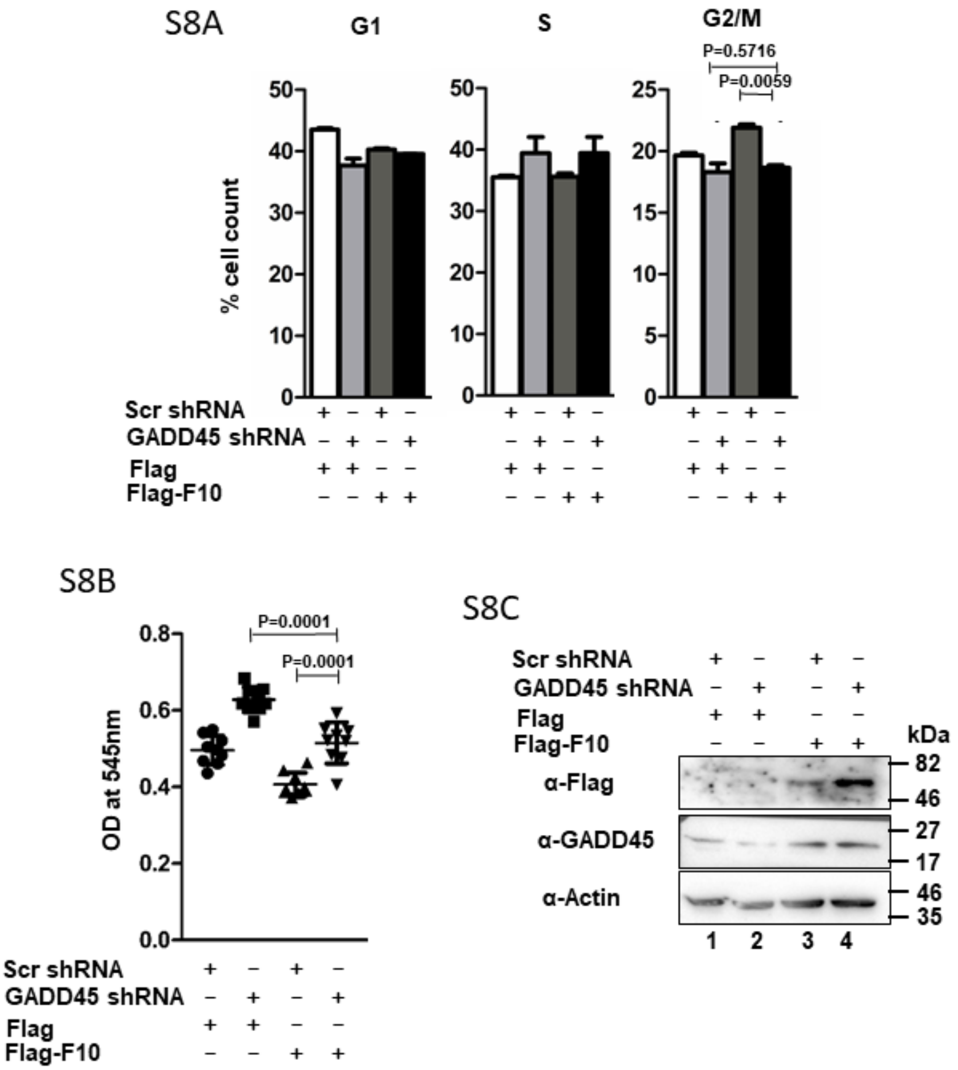

Figure S8: RASSF10 was expressed in AGS cells with or without GADD45a knockdown and analyzed the status of cell cycle profiles by Flow cytometry analysis (A) and cell proliferation/viability using MTT assay (B). (C) Expression levels of RASSF10 and efficiency of GADD45a knockdown were determined by western blot analysis using indicated antibodies. β-actin was used as loading control (n=3 and data is expressed as mean ± SD). RASSF10 referred as F10; Cyclin-B referred as Cyc B.

Supplementary Figure 9

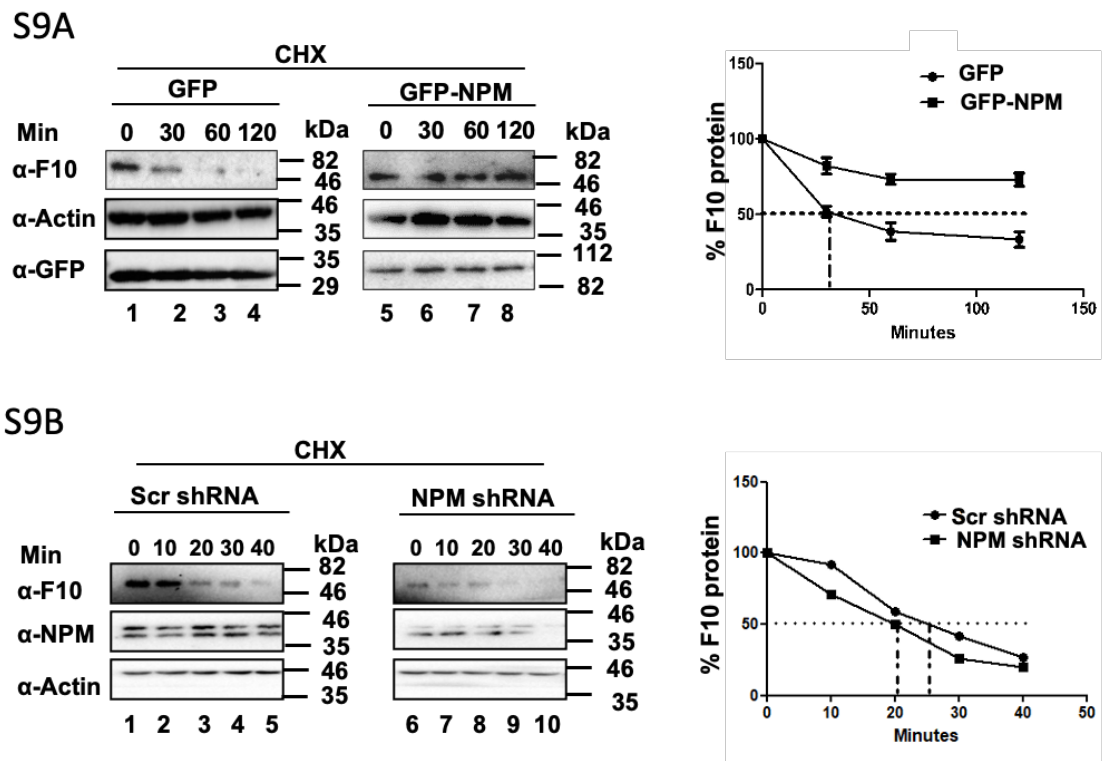

Figure S9: (A) Results from cycloheximide (CHX) chase assay indicate that the expression of NPM increased the steady state levels of RASSF10 whereas (B) Knockdown of NPM resulted in decreased half-life of RASSF10. The densitometry analyses were carried out by normalizing to β-actin and plotted against time to calculate half-life of RASSF10 (n=3 and data is expressed as mean ± SD). RASSF10 referred as F10.

Supplementary Figure 10

S10A

BDM-PUB: Prediction of Ubiquitination sites with Bayesian Discriminant Method

Peptide	Position	Score	Threshold
**MDPSEKKISVWIC	6	0.95	0.30
SLAMTQEKQRRVVRK	172	0.34	0.30
KQRRVVRKAFRKLAK	179	1.57	0.30
VVRKAFRKLAKLNRR	183	3.51	0.30
KAFRKLAKLNRRRQQ	186	1.91	0.30
NTDLEAVKSDLDYSQ	412	1.29	0.30
DQARGLAKSGPGNDE	476	1.00	0.30

UbPred: predictor of protein ubiquitination sites

MDPSEKKISVWICQEEKLVSGLSRRTTCSDVVRVLLEDGCRRRRRQRRSRRLGSAGDPHGPGELPEPPNEDDEDDDEALPQGML
CGPPQCYCIVEKWRGFERILPNKTRILRLWAAWGEEQENVRFVLVRSEASLPNAGPRSAEARVVLSRERPCPARGAPARPSLAMT
QEKQRRVVRKAFRKLAKLNRRRQQQTPSSCSSTSSSTASSCSSSPRTHESASVERMETLVHLVLSQDHTIRQQVQRLHELDREIDH
YEAKVHLDRMRRHGVNYVQDTYLVGAGIELDGSRPGEEPEEVAAEAEEAAAAPPLAGEAQAAALEELARRCDDLLRLQEQRVQQE
ELLERLSAEIQEELNQRWMRRRQEELAAREEPLEPDGGPDGELLLEQERVRTQLSTSLYIGLRLNTDLEAVKSDLDYSQQQWDSKK
RELQGLLQTLHTLELTVAPDGAPGSGSPSREPGPQACADMWVDQARGLAKSGPGNDEDSDTGLSSMHSQDSDSLPMCESLV

Label	Score range	Sensitivity	Specificity
Low confidence	0.62 ≤ s ≤ 0.69	0.464	0.903
Medium confidence	0.69 ≤ s ≤ 0.84	0.346	0.950
High confidence	0.84 ≤ s ≤ 1.00	0.197	0.989

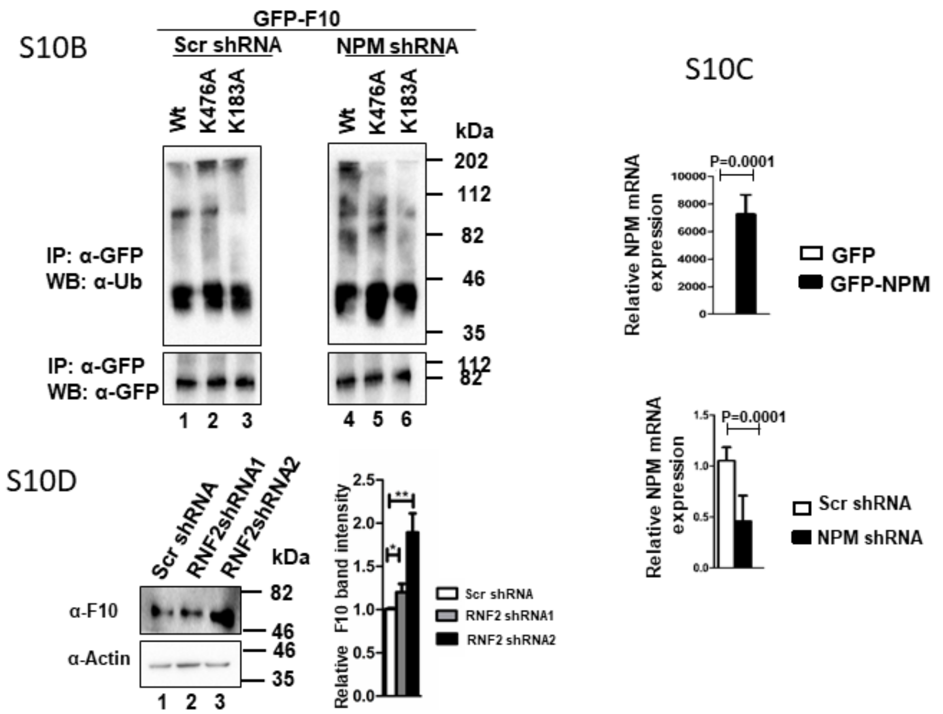

Figure S10: (A) Bioinformatics analysis predicted lysine residues at positions 183 and 476 may be the potential ubiquitination sites on RASSF10 protein. (B) NPM knockdown promotes the polyubiquitination of wild type and K476A not K183A variant of RASSF10. (C) Expression and knockdown efficiency of NPM were analysed by RT-qPCR. (D) RASSF10 protein levels were increased with knockdown of *RNF2* in AGS cells. The densitometry analysis of western blots was carried out by normalizing the expression level of endogenous proteins to β-actin, as loading control (n=3 and data is expressed as mean ± SD). RASSF10 referred as F10; Ubiquitin referred as Ub. (*p<0.05; **p<0.01).

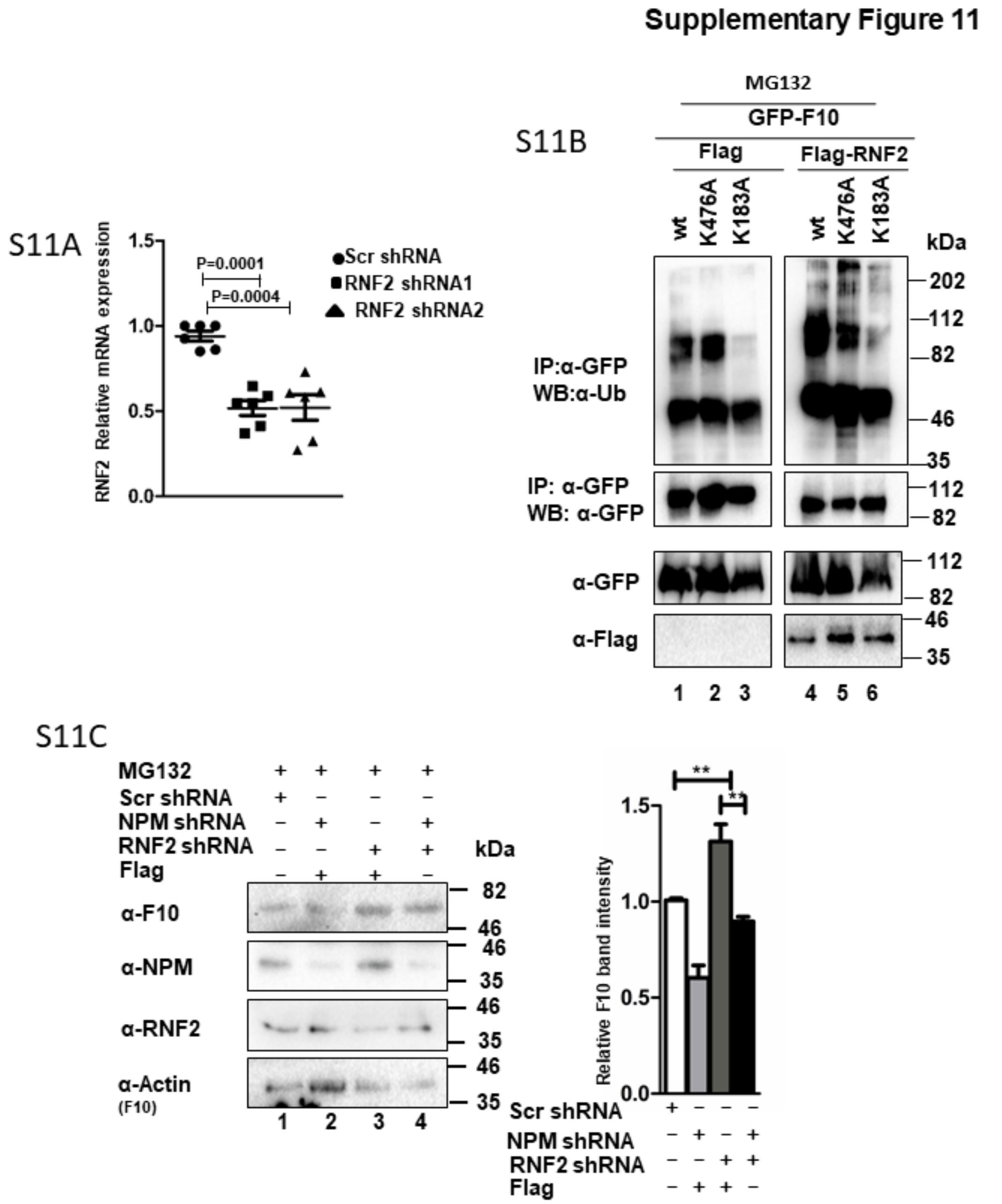

Figure S11: (A) RT-qPCR analysis was used to determine the efficiency of *RNF2* knockdown in AGS cells. (B) RNF2 expression induces polyubiquitination of wildtype and K476A not K183A variant of RASSF10. (C) Western blot analysis was performed to check the effect of NPM and RNF2 knockdowns on RASSF10 protein levels. The densitometry analysis of western blots was carried out by normalizing the expression level of endogenous proteins to β-actin, as loading control (n=3 and data is expressed as mean ± SD). RASSF10 referred as F10; Ubiquitin referred as Ub. (*p<0.05; **p<0.01).

Supplementary Table S1: List of differentially expressed proteins in presence of RASSF10 expression were identified by LC-MS/MS.

Spot ID	Accession No.	Description	Score	Coverage	No. of Peptides	PSMs	No. of AAs	MW [kDa]	Calc. pI	MW [kDa] 2D	pI 2D
2	P02769	Serum albumin	38.57	22.41	12	16	607	69.2	6.18	60	5.4
4	P48616	Vimentin	62.86	37.98	19	21	466	53.7	5.12	54	5
6	P04181	Ornithine aminotransferase	85.30	43.51	14	25	439	48.5	7.03	47	6.4
8	P31942	Heterogeneous nuclear ribonucleoprotein H3	305.44	63.87	15	85	346	36.9	6.87	35	6.5
10	P31942	Heterogeneous nuclear ribonucleoprotein H3	129.64	64.16	13	37	346	36.9	6.87	35	6.5
12	P06748	Nucleophosmin	4420.53	62.24	23	1150	294	32.6	4.78	32	4
15	P04406	Glyceraldehyde-3-phosphate dehydrogenase	1059.90	59.10	16	247	335	36.0	8.46	30	8.2
18	Q6PUJ7	Prohibitin	2682.29	94.85	28	653	272	29.8	5.76	25	5.2
19	Q13162	Peroxiredoxin-4	841.92	46.49	17	237	271	30.5	6.29	23	5.7
37	P05387	60S acidic ribosomal protein	1668.76	80.00	12	428	115	11.7	4.54	13	3.5
38	P78371	T-complex protein 1 subunit beta	3229.10	73.08	42	889	535	57.5	6.46	41	7
39	P12277	Creatine kinase B-type	90.37	47.77	11	24	381	42.6	5.59	33	6.8
40	P68032	Actin, alpha cardiac muscle 1	315.01	31.83	14	86	377	42	5.39	30	6.4
41	P07195	L-lactate dehydrogenase B chain	1730.66	70.96	30	522	334	36.6	6.05	27	6
44	H0YN26	Acidic leucine-rich nuclear phosphoprotein32 family member A	82.38	40.11	6	27	177	20.0	4.58	18	5.5
45	E7EUT5	Glyceraldehyde-3-phosphate dehydrogenase	425.97	45.38	7	118	260	27.9	6.95	20	6.5
46	F8W1A4	Adenylate kinase 2, mitochondrial	48.33	45.26	7	12	232	25.6	7.83	21	8.4
46	J3QSB7	Purine nucleoside phosphorylase	46.62	29.34	4	13	242	26.8	6.84	21	8.4
51	B5BU83	Stathmin	113.45	48.32	8	43	149	17.3	5.97	16	5.7
53	P06733	Alpha-enolase	2777.69	81.8	41	797	434	47.1	7.39	41	8.5
55	E7EUT5	Glyceraldehyde-3-phosphate dehydrogenase	41.12	63.08	8	11	260	27.9	6.95	29	8
56	B2R860	Ribose-phosphate pyrophosphokinase	59.41	21.38	5	15	318	34.6	6.46	27	7.2
58	P53004	Biliverdin reductase A	64.67	43.24	9	16	296	33.4	6.44	30	7
66	P63104	14-3-3 protein zeta/delta	236.98	56.73	14	71	245	27.7	4.79	23	3
67	P54105	Methylosome subunit pICln	1274.44	54.01	7	384	237	26.2	4.11	29	3
69	Q14257	Reticulocalbin-2	529.60	59.31	18	153	317	36.9	4.40	40	3.5
70	Q5T8M8	Actin, alpha skeleton muscle	98.59	26.48	9	28	287	32.0	5.41	32	3
71	Q01105	Protein SET	283.32	53.79	22	75	290	33.5	5.24	31	3.5
72	O14602	Eukaryotic translation initiation factor 1A,	55.55	37.5	7	17	144	16.4	5.24	16	4.2
72a	P13639	Elongation factor 2	1119.17	50	45	334	858	95.3	6.83	80	7.9
73a	P13639	Elongation factor 2	576.88	40.09	27	151	858	95.3	6.83	80	8
74	K7EM73	Calpain small subunit 1 (Fragment)	33.55	63.19	4	8	163	15.9	4.91	22	4.2
75	P35232	Prohibitin	84.79	64.71	14	25	272	29.8	5.76	22	6.3
76	P62258	14-3-3 protein epsilon	52	46.67	10	15	255	29.2	4.74	23	6.1
78	Q13347	Eukaryotic translation initiation factor 2 subunit	341.42	58.46	18	99	325	36.5	5.64	28	5.3
79	Q15181	Inorganic pyrophosphatase	1061.49	60.9	24	275	289	32.6	5.86	28	5.5
79	P07195	L-lactate dehydrogenase B chain	227.41	49.1	21	67	334	36.6	6.05	28	5.5
81	B3KX11	cDNA FLJ44436 fis, clone UTERU2019706, highly similar to T-complex protein 1 subunit gamma	1022.60	56.51	34	294	522	57.9	6.93	55	7
84	Q5T6W5	Heterogeneous nuclear ribonucleoprotein K	712.63	43.22	21	195	428	47.5	5.63	42	5.5
85	P31930	Cytochrome b-c1 complex subunit 1,	651.62	35.42	18	181	480	52.6	6.37	41	6.1
87	P63104	14-3-3 protein zeta/delta	61.72	42.04	11	17	245	27.7	4.79	21	5
88	P04632	Calpain small subunit 1	303.29	52.99	9	76	268	28.3	5.2	20	4.7
89	P09211	Glutathione S-transferase P	138.93	57.14	7	40	210	23.3	5.64	20	5
90	Q04760	Lactoylglutathione lyase	336.63	44.57	12	99	184	20.8	5.31	19	4.8
91	P12004	Proliferating cell nuclear antigen	177.82	43.30	7	51	261	28.8	4.69	27	3.9
92	E7EUT5	Glyceraldehyde-3-phosphate dehydrogenase	41.40	41.92	5	11	260	27.9	6.95	29	7
93	P04406	Glyceraldehyde-3-phosphate dehydrogenase	1782.62	59.40	18	504	335	36.0	8.46	30	9
94	P04406	Glyceraldehyde-3-phosphate dehydrogenase	2917.44	75.22	22	772	335	36	8.46	28	10
96	Q53HF2	Heat shock 70kDa protein B isoform 2 variant	70.46	35.5	13	19	493	53.5	5.86	48	6.2
97	Q5CAQ5	Tumor rejection antigen (Gp96) 1	220.31	53.12	38	67	802	92.3	4.86	90	3.8

¶¶ The table shows list of 50 significantly deregulated proteins in presence of RASSF10. Details of Spot ID, accession number, protein name, score, % coverage, number of covering peptides, peptide spectrum match (PSM), amino acid number (AA), molecular weight (calc MW) and calculated isoelectric point (calc. pI) are detailed.

Supplementary Table S2: RT-qPCR analysis validated the expression of genes that are deregulated by RASSF10.

Spot ID	Accession No.	Description	Score	Coverage	Relative Status in Mass spec analysis	qRT PCR	
						Status	P-Value
10	P31942	Heterogeneous nuclear ribonucleoprotein H3	129.64	64.16	Up	Up	0.04
12	P06748	Nucleophosmin	4420.53	62.24	Up	Up	0.034
15	P04406	Glyceraldehyde-3-phosphate dehydrogenase	1059.90	59.10	Down	Up	0.028
18	Q6PUJ7	Prohibitin	2682.29	94.85	Up	Up	0.6
19	Q13162	Peroxiredoxin-4	841.92	46.49	Up	Up	0.8
37	P05387	60S acidic ribosomal protein	1668.76	80.00	Down	Nc	0.3
38	P78371	T-complex protein 1 subunit beta	3229.10	73.08	Down	Down	0.05
39	P12277	Creatine kinase B-type	90.37	47.77	Down	Down	0.4
40	P68032	Actin, alpha cardiac muscle 1	315.01	31.83	Down	Nc	0.2
41	P07195	L-lactate dehydrogenase B chain	1730.66	70.96	Down	Down	0.02
44	H0YN26	Acidic leucine-rich nuclear phosphoprotein32 family member A	82.38	40.11	Down	Nc	0.19
46	F8W1A4	Adenylate kinase 2, mitochondrial	48.33	45.26	Down	Down	0.08
46	J3QSB7	Purine nucleoside phosphorylase	46.62	29.34	Down	Up	0.4
51	B5BU83	Stathmin	113.45	48.32	Down	Up	0.54
53	P06733	Alpha-enolase	2777.69	81.8	Down	Up	0.021
56	B2R860	Ribose-phosphate pyrophosphokinase	59.41	21.38	Down	Down	0.05
58	P53004	Biliverdin reductase A	64.67	43.24	Down	Up	0.14
66	P63104	14-3-3 protein zeta/delta	236.98	56.73	Down	Up	0.7
67	P54105	Methylosome subunit pICln	1274.44	54.01	Down	Nc	0.84
69	Q14257	Reticulocalbin-2	529.60	59.31	Down	Down	0.16
72	O14602	Eukaryotic translation initiation factor 1A,	55.55	37.5	Up	Up	0.06
73a	P13639	Elongation factor 2	576.88	40.09	Up	Up	0.042
74	K7EM73	Calpain small subunit 1 (Fragment)	33.55	63.19	Up	Nc	0.62
76	P62258	14-3-3 protein epsilon	52	46.67	Down	Nc	0.91
78	Q13347	Eukaryotic translation initiation factor 2 subunit	341.42	58.46	Down	Up	0.07
79	Q15181	Inorganic pyrophosphatase	1061.49	60.9	Down	Nc	0.75
85	P31930	Cytochrome b-c1 complex subunit 1,	651.62	35.42	Down	Up	0.08
87	P63104	14-3-3 protein gamma	61.72	42.04	Down	Down	0.067
89	P09211	Glutathione S-transferase P	138.93	57.14	Down	Up	0.05
90	Q04760	Lactoylglutathione lyase	336.63	44.57	Down	Down	0.32
91	P12004	Proliferating cell nuclear antigen	177.82	43.30	Down	Nc	0.4
96	Q53HF2	Heat shock 70kDa protein 1 isoform 2 variant	70.46	35.5	Down	Up	0.04
97	Q5CAQ5	Tumor rejection antigen (Gp96) 1	220.31	53.12	Down	Down	0.5

Supplementary Table S3: Details of primers and shRNA sequence used in the current investigation.

Gene	Sequence 5' to 3'
RASSF10 Fw<br>RASSF10 Rv	GCTCAGTACCAGCCTTTACA<br>ACCGTCAGCTCCAAAGTGT
GADD45A Fw<br>GADD45A Rv	CCCGATAACGTGGTGTTG<br>CAGGATGTTGATGTCGTCGTTCT
Actin Fw<br>Actin Rv	CCTTGCACATGCCGGAG<br>GCACAGAGCCTTCGCCTT
NPM Fw<br>NPM Rv	GGTCTGCCCCTGGAGGT<br>GGCGCTTTTTCTTCAGCTT
FBW7 Fw<br>FBW7 Rv	CCAACTCTCCTCCCCATTCT<br>TGCTGAACATGGTACAAGCC
SKP2 Fw<br>SKP2 Rv	CCAGGAACTGCTCTCAAACC<br>GAAGGGAGTCCCATGAAACA
FBXO32 Fw<br>FBXO32 Rv	AAAGAGCGCCATGGATATTG<br>TCAGGGATGTGAGCTGTGAC
STUB1 Fw<br>STUB1 Rv	AGCAGGGCAATCGTCTGTT<br>TGCTGCTGCATCTTCAGGTA
DDB1 Fw<br>DDB1 Rv	GATCATCCGGAATGGAATTG<br>TGAGAACTCTTGTCTGGCCC
UBR7 Fw<br>UBR7 Rv	GAGATTTGCTGATGAAGGCA<br>GGCTTGAATGAGAAAGCTTCA
RNF138 Rw<br>RNF138 Rv	AGATGATTTCTACTGCCCCG<br>TGAGTTCCGTTTGTCTGCAC
RNF2 FW<br>RNF2 Rv	AAGCTGAGGCTCGCCATATT<br>TGAGTTCCGTTTGTCTGCAC
UBR3 Fw<br>UBR3 Rv	GGCTAGAGAGAGGCAGCAGA<br>TACTGCCTCAGAAACCTGTG
URF1 Fw<br>URF1 Rv	ACCAAGGTGGAGGAGCTGAG<br>AGGAGCTGGATGGTGTCATT
URF2 Fw<br>URF2 Rv	TTGCTGCTGATGAAGACGTT<br>CCATTACCACATCACCAACAT
CYCLINB1 Fw<br>CYCLINB1 Rv	CCAAAGGCCACTAGGCCT<br>GGGGCGGGGCCACAG
HNRPH3 Fw<br>HNRPH3 Rv	CCTTCTTTGAACTGGACCCC<br>CTGTCCCATCACTAGCGTCA
HSPB1 Fw<br>HSPB1 Rv	TGACGGTCAAGACCAAGGAT<br>GGACAGGGAGGAGGAAACTT
PHB Rw<br>PHB Rv	ACCTCGAAAAATCTCCTCCC<br>CTCGTTCCTCGTAGTCCAGC
GAPDH Fw<br>GAPDH Rv	TTAAAAGCAGCCCTGGTGAC<br>CTCTGCTCCTCCTGTTCGAC
GSTP1 Fw<br>GSTP1 Rv	GACCTCCGCTGCAAATACAT<br>AATGAAGGTCTTGCCTCCCT
LDHB Fw<br>LDHB Rv	CACCAGTTGCGGAAGAAGA<br>CCAAAACATCCACAAGAGCA
LMNB1 Fw<br>LMNB1 Rv	GATCGAGCTGGGCAAGTG<br>TGCAGTAGCAAGAGCTGCAT
ANP32A Fw<br>ANP32A Rv	GGATTCATTTAGAGCTGCGG<br>GGTGAGGCCTACGTTGATTG
CAPNS1 Fw<br>CAPNS1 Rv	TCCGACGCTACTCAGATGAA<br>AGCCACTCCTGGATGTTCAC
CCT2 Fw<br>CCT2 Rv	GTGTGGAACGCCTAGCTCTT<br>GCAACCCCAGAAAAGTGAAT
Cox6A Fw<br>Cox6A Rv	AGCCAGTTGGAAGTGGATTC<br>TGTACCTGAAGTCGCACCAC
ACTRIA1 Fw<br>ACTRIA1 Rv	TCCCCAGAAGGAGAGATTCC<br>TTGGAAAGCAGTATTTGGGG

AK2 Fw<br>AK2 Rv	GGCAGAACCCGAGTATCCTA<br>CTCAGCATGTCCCCAGTAGC
CDK1 Fw<br>CDK1 Rv	TTTCATGGCTACCACTTGACC<br>TAAGCCGGGATCTACCATACC
ENO Fw<br>ENO Rv	GAGATCTCGCCGGCTTTAC<br>AGTCAAAGATCTCCCTGGCA
EEF2 Fw<br>EEF2 Rv	AGTCGGGAGAGCACATCATC<br>CGACTCTTCACTGACCGTCTC
**Cloning Primers:**	
RASSF10 Fw<br>RASSF10 Rv	ATCGTGGGGTACCATGGATCCTTCGG<br>GCCTCACATATGCTACACAAGGGATTCG
$RASSF10^{K183A}$ Fw<br>$RASSF10^{K183A}$ Rv	GCCTTTCGCGCGCTGGCCAAGCTC<br>GAGCTTGGCCAGCGCGCGAAAGGC
$RASSF10^{K476A}$ Fw<br>$RASSF10^{K476A}$ Fw	GGACTGGCCGCTAGCGGTCCTGGC<br>GCCAGGACCGCTAGCGGCCAGTCC
RNF2 Fw<br>RNF2 RV	AGTCGTGGTACCATGTCTCAGGCTGTGC<br>TTAGCCCTCGAGTCATTTGTGCTCCTTTGTAGG
**shRNA sequence:**	
GADD45a	CCGGGAAGACCGAAAGGATGGATAACTCGAGTTATCCATCCTTTCGGTCTTCTTTTTG
NPM	CCGGGCGCCAGTGAAGAAATCTATACTCGAGTATAGATTTCTTCACTGGCGCTTTTTG
p^{53}	CCGGCGGCGCACAGAGGAAGAGAATCTCGAGATTCTCTTCCTCTGTGCGCCGTTTTT
RASSF10	CCGGAGAATGTGCGCTTCGTGCTAGCTCGAGCTAGCACGAAGCGCACATTCTTTTTTG
RNF2	CCGGGCCAGGATCAACAAGCACAATCTCGAGATTGTGCTTGTTGATCCTGGCTTTTTG
Scrambled	CCGGGCGCGATAGCGCTAATAATTTCTCGAGAAATTATTAGCGCTATCGCGCTTTTT

Supplementary Table S4: Details of reagents and antibodies used in the current investigation**.**

Reagent	Source	Identifier
**Antibodies:**		
Mouse monoclonal anti-GFP	Santa cruz	Catalog: Sc996
Rabbit polyclonal anti-RASSF10	Abcam	Catalog: Ab113105
Mouse monoclonal anti-Actin	Sigma	Catalog:A5441
Mouse monoclonal anti-Flag	Sigma	Catalog: F3165
Rabbit polyclonal anti-Flag	Sigma	Catalog: F7425
Rabbit polyclonal anti-CyclinB	Santa cruz	Catalog: Sc166152
Mouse monoclonal anti-Cdk1	Abcam	Catalog: Ab18
Mouse monoclonal anti-RNF2	Sigma	Catalog: WH0006045M1
Mouse monoclonal anti-Ubiquitin	Cell Signalling Technology	Catalog: 3936S
Rabbit polyclonal anti-NPM1	Santa cruz	Catalog: Sc5564
Mouse monoclonal anti-NPM1	Abcam	Catalog: Ab15440
Rabbit polyclonal anti-GADD45a	Cell Signalling Technology	Catalog: 4632S
Mouse monoclonal anti-Lamin	Santa Cruz	Catalog: Sc377000
Rabbit polyclonal anti-GAPDH	Cell Signalling Technology	Catalog: 2118S
Rabbit polyclonal anti-APC1	Abcam	Catalog: ab133397
Rabbit polyclonal anti-APC1p355	Abcam	Catalog: ab10923
Goat anti-Mouse	Bio-rad	Catalog:1706516
Goat anti-Rabbit	Bio-rad	Catalog: 1706515
**Chemicals:**		
Dullecco's Minimum Essential Medium (DMEM)	GIBCO	Catalog: 12800-017
Trypsin EDTA	GIBCO	Catalog: 15400-054
Antibiotic-Antimycotic	GIBCO	Catalog: 15240-062
Fetal Bovine Serum (FBS)	GIBCO (south America)	Catalog: 10270-106
Phosphate Buffer Saline (PBS)	GIBCO	Catalog: 21600-010
Polyethylenimine (PEI)	Polysciences	Catalog: 23966-1
Dimethyl Sulfoxide (DMSO)	Sigma-Aldrich	Catalog: D8414
Proteosome inhibitor, MG132	Sigma-Aldrich	Catalog: M7449
TRIzol Reagent	Takara Clontech	Catalog: 9101
3-(4,5-Dimethylthiazol-2-yl)-2,5-Diphenyltetrazolium Bromide (MTT)	Sigma-Aldrich	Catalog: M2003
Propidium Iodide(PI)	Sigma-Aldrich	Catalog: P4170
AnnexinV-FITC apoptosis detection kit	BD Biosciences	Catalog: 556547
BrdU	BD Biosciences	Catalog: 51-9000019AK
Syber green	Takara Clontech	Catalog: RR820
Thymidine	Sigma	Catalog: T79250
IPG strip pH3-11 NL, 11cm	GE Health care	Catalog: 17600374
IPG strip pH4-7, 7cm	GE Health care	Catalog: 17600110
Mycoplasma detection kit	Sigma-Aldrich	Catalog: MP0035

Supplementary Table S5: Details of gastric cancer tissue samples used for RT-qPCR analysis.

Sl.No:	Clinicopathological characteristic	Number of samples
**1**	**Total**	**16**
**2**	**Gender:**<br>Male<br>Female	 15<br>1
**3**	**Age (years):**<br>≤59<br>>59	 7<br>9
**4**	**Grade of differentiation:**<br>Low (I-II)<br>Middle (III)<br>High (IV)	 8<br>3<br>5

Supplementary Movie 1: AGS cells were ectopically expressed with GFP-RASSF10 and were synchronized at G1 stage by double thymidine block and the status of cell cycle was analysed using live cell imaging as described in Materials and Methods.

Supplementary Movie 2: AGS cells were ectopically expressed with GFP and were synchronized at G1 stage by double thymidine block and the status of cell cycle was analysed using live cell imaging as described in Materials and Methods.
